# A glimpse on the pattern of rodent diversification: a phylogenetic approach

**DOI:** 10.1186/1471-2148-12-88

**Published:** 2012-06-14

**Authors:** Pierre-Henri Fabre, Lionel Hautier, Dimitar Dimitrov, Emmanuel J P Douzery

**Affiliations:** 1 Center for Macroecology, Evolution and Climate (CMEC, Department of Biology), Zoological Museum, University of Copenhagen, Universitetsparken 15, DK-2100 Copenhagen, Denmark; 2 Institut des Sciences de l’Evolution (ISEM, UMR 5554 CNRS-IRD), Université Montpellier II, Place E. Bataillon - CC 064 - 34095 Montpellier Cedex 5, France; 3 Department of Zoology, University of Cambridge, Cambridge CB2 3EJ, UK

## Abstract

**Background:**

Development of phylogenetic methods that do not rely on fossils for the study of evolutionary processes through time have revolutionized the field of evolutionary biology and resulted in an unprecedented expansion of our knowledge about the tree of life. These methods have helped to shed light on the macroevolution of many taxonomic groups such as the placentals (Mammalia). However, despite the increase of studies addressing the diversification patterns of organisms, no synthesis has addressed the case of the most diversified mammalian clade: the Rodentia.

**Results:**

Here we present a rodent maximum likelihood phylogeny inferred from a molecular supermatrix. It is based on 11 mitochondrial and nuclear genes that covers 1,265 species, *i.e.*, respectively 56% and 81% of the known specific and generic rodent diversity. The inferred topology recovered all Rodentia clades proposed by recent molecular works. A relaxed molecular clock dating approach provided a time framework for speciation events. We found that the Myomorpha clade shows a greater degree of variation in diversification rates than Sciuroidea, Caviomorpha, Castorimorpha and Anomaluromorpha. We identified a number of shifts in diversification rates within the major clades: two in Castorimorpha, three in Ctenohystrica, 6 within the squirrel-related clade and 24 in the Myomorpha clade. The majority of these shifts occurred within the most recent familial rodent radiations: the Cricetidae and Muridae clades. Using the topological imbalances and the time line we discuss the potential role of different diversification factors that might have shaped the rodents radiation.

**Conclusions:**

The present glimpse on the diversification pattern of rodents can be used for further comparative meta-analyses. Muroid lineages have a greater degree of variation in their diversification rates than any other rodent group. Different topological signatures suggest distinct diversification processes among rodent lineages. In particular, Muroidea and Sciuroidea display widespread distribution and have undergone evolutionary and adaptive radiation on most of the continents. Our results show that rodents experienced shifts in diversification rate regularly through the Tertiary, but at different periods for each clade. A comparison between the rodent fossil record and our results suggest that extinction led to the loss of diversification signal for most of the Paleogene nodes.

## Background

A fundamental question in evolutionary biology is understanding why some clades are highly diverse. Species diversity is the result of the balance between speciation and extinction whereas morphological disparity is primarily a consequence of adaptation [1-3]. For a long time, only the study of the fossil record provided a direct view of the patterns of diversification revealing major speciation and extinction trends through time [4]. Development of methods that do not rely on fossils for the study of the evolutionary processes through time [5] have dramatically changed the way we study diversification patterns. The use of dated phylogenetic trees in combination with knowledge on species assemblies allows us to *(1)* estimate speciation and extinction rates [6], *(2)* detect shifts in diversification rates [7,8], *(3)* test diversification constancy through time [9,10] and *(4)* study the link between biological traits and diversification within clades [11,12].

Throughout the Cenozoic, rodents underwent an extraordinary adaptive radiation. As a result rodents represent nearly half of the current mammalian diversity with more than 2,261 species organized into 474 genera [13]. These small to medium-sized placentals have spread over all continents (except Antarctica) and most islands, where they occupy virtually all terrestrial ecosystems from tropical rainforests and deserts to the arctic tundra. New species and genera are being described each year, such as *Laonastes aenigmamus*[14], the sole extant representative of a morphologically and phylogenetically distinct family, the Diatomyidae [15,16]. Rodents also display a wide range of life histories and ecomorphological adaptations including fossorial, arboreal, subaquatic, jumping and gliding capacities. Their outstanding diversity among mammals, combined with the richness of their fossil record, makes rodents a suitable model to study the factors that promote morphological diversity and trigger evolutionary radiations.

Repetitive bursts of speciation and a high level of homoplasy in morphological characters [17-19] have hindered delimitation of inter- and intra-familial relationships within rodents. However, a resurgence of interest in rodent phylogeny using molecular markers, most notably mitochondrial markers [20-23], nuclear genes [16,24-30] and retroposed elements [31], has provided a new insight of familial relationships and has challenged traditional classifications based on myological (*e.g.*[32], [33] and [18]) and cranio-dental characters [34]. Simultaneously, many molecular studies have addressed phylogenetic relationships at lower taxonomic levels, releasing a large number of sequences from a variety of loci [21,35-54]. These recent developments make now possible the construction of a large phylogeny based on DNA data for this mammalian order. Such a phylogenetic framework is the basis for macroevolutionary and comparative meta-analyses that aim to address questions about rodent evolutionary history.

Two approaches have been proposed to reconstruct large evolutionary trees from partially overlapping character and taxon datasets: the supertree, and the supermatrix. In the supertree approach, independent data sets are analysed separately to yield source topologies which are subsequently combined to produce a larger phylogenetic tree [55,56]. In contrast, supermatrix analyses use characters gathered from the widest possible range of taxa in a single analysis to provide a “large tree”. Gatesy *et al.*[57,58] compared the two approaches and brought attention to the methodological constraints of the supertree approach e.g. *(i)* source data which contain non-cladistic characters such as taxonomy lists, *(ii)* duplication of homologous characters or *(iii)* robustness values of tree nodes that are difficult to interpret. Gatesy *et al.*[58] supported the use of a supermatrix as the combination of independent features could reveal hidden relationships [59]. To date, the only large-scale comprehensive phylogeny available for rodents is part of the family-level supertree of Beck *et al.*[60], and the species-level supertree published by Bininda-Emonds *et al.*[61] which included nearly all extant families and species of mammals. Furthermore due to lack of phylogenetic data for many of the rodent groups at that time, their final topologies contain a large amount of polytomies (less than < 40% of the branches are fully resolved at the genus level) and do not reflect our current knowledge of rodent systematics. We therefore expect that a more robust framework for rodent molecular phylogeny may benefit from a gene concatenation approach as illustrated by the family-level supermatrix tree of Meredith *et al.*[62]. Here, we present the first large-scale phylogenetic analysis which includes the most representative molecular markers for rodents. The inferred topology is subsequently used to provide divergence date estimates with a relaxed molecular clock. Our species level rodent phylogeny allows us to address specifically the following questions: *(1)* Is the rate of diversification constant over all lineages ? *(2)* Within which lineages, if any, do shifts in diversification rate occur ? *(3)* When did major rodent diversification events occur during the Tertiary ? *(4)* Can we connect potential shifts in diversification rate to macroevolutionary events ?

## Results and Discussion

### Phylogenetic results and systematics

**Figure 1:**
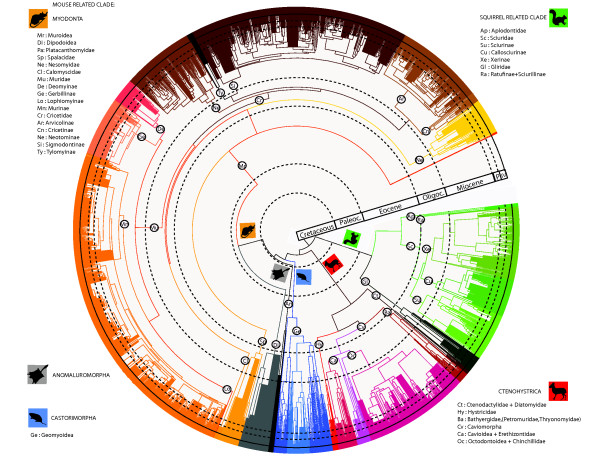
**Rodent species-level evolutionary dated tree.** The species-level chronogram is based on the ML topologies from 8 supermatrix trees. Stratigraphic scale : P : Paleocene, E : Eocene, O : Oligocene, M : Miocene, P : Pliocene, Pl : Pleistocene. Bootstrap and divergence time estimates for all nodes are detailed in Supplementary Data.

**Figure 2:**
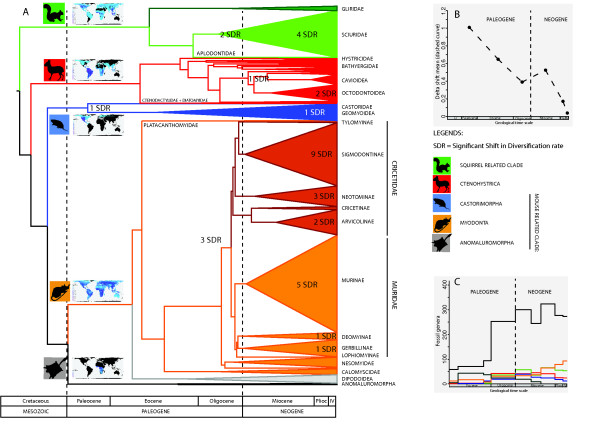
**Diversification of rodents through time.** Left part **(A)**: Simplified family-level phylogenetic dated tree of rodents. Stratigraphic scale is in the lower part. Significant shifts in diversification rate (SDR) are indicated (see also Table 1 and Additional file 2: Figure S2, Additional file 3: Figure S3, Additional file 4: Figure S4, Additional file 5: Figure S5, Additional file 6: Figure S6, Additional file 7: Figure S7, Additional file 8: Figure S8, Additional file 9: Figure S9, Additional file 10: Figure S10, Additional file 11: Figure S11, Additional file 12: Figure S12 to Additional file 13: Figure S13). Upper right part **(B)**: Variation through each epoch bin of the mean of absolute nodal *Δ*shift statistics values calculated from the overall 2,263-taxon topology. Lower right part **(C)**: Histogram of the number of rodents genera through Tertiary (McKenna and Bell, 1997). This illustrates the evolution of genus diversity for all rodents (black), extinct stem rodents (darkgreen), Muroidea+Anomaluroidea (orange), Castorimorpha (blue), Sciuroidea (green) and Ctenohystrica (red).

Since the first mammal supertree [63], there has been no integrated, molecular-based synthesis of rodent systematics. However, several extensive mitochondrial molecular studies of other mammalian orders have been performed for Primates [64-66], Carnivora [67], Cetartiodactyla [68,69] and Chiroptera [70]. The molecular supermatrix presented here is the first attempt to include all Rodentia taxa for which mitochondrial and nuclear DNA sequences are available in public databases within a common phylogenetic framework. The supermatrix concatenates 11 genes, and contains 1,265 taxon sequences aligned for 15,535 sites, with 75% of missing character states. Maximum likelihood analysis yields a phylogenetic hypothesis for Rodentia, with bootstrap values (BP) greater than 70% for 64% of the nodes summarized in Figures 1 and 2 (see also Additional file 1: Figure S1, Additional file 2: Figure S2, Additional file 3: Figure S3, Additional file 4: Figure S4, Additional file 5: Figure S5, Additional file 6: Figure S6, Additional file 7: Figure S7, Additional file 8: Figure S8, Additional file 9: Figure S9, Additional file 10: Figure S10, Additional file 11: Figure S11, Additional file 12: Figure S12, Additional file 13: Figure S13 for details about the species-level topology). Figure 1, Figure 2 and Additional file 1: Figure S1 represents a topological summary of all other topological figures.

**Table 1:** Rodent sister-clades with significant (P < 0.05) and marginal (0.05 < P < 0.10) shifts in diversification rate using _*Δ*1_shift statistics

**Clade**	_***Δ*1**_	**P-value**
CTENOHYSTRICA		
(1) Bathyergidae: **Heliophobius** / ***Bathyergus,Cryptomys*Clade**	1.99	0.09
(2) Caviomorpha: Dasyproctidae / **Caviidae**	2.04	0.06
(3) Octodontoidea: Base of **Octodontidae**+**Ctenomyidae** / **Echimyidae**	2.40	**0.05**
(4) Echimyidae : **Base *Proechimys***	2.75	**0.05**
(5) Ctenomyidae: *Ctenomys leucodon***/ Sister clade**	3.46	**0.01**
(6) Ctenomyidae: *Ctenomys maulinus*clade / **Sister clade**	2.23	0.06
(7) Ctenomyidae: *Ctenomys mendocinus* / **Sister clade**	2.54	0.06
SCIUROIDEA		
(8) Sciuridae: Aplodontidae / **Sciuridae**	2.25	**0.05**
(9) Sciuridae: *Sciurillus* / **other Sciuridae**	5.36	**0.01**
(10) Sciuridae: **Flying squirrel clade** (Pteromyini tribe)	2.82	**0.01**
(11)Sciuridae: *Nannosciurus* / **Sister clade**	3.28	**0.02**
(12) Sciuridae: *Dremomys* clade / ***Callosciurus* clade**	3.28	**0.03**
(13) Sciuridae:*Paraxerus clade* / **(*Tamias*, *Spermophilus*) clade**	1.73	0.08
(14) Sciuridae: *Protoxerus* clade / ***Funisciurus*clade**	1.96	0.07
(15) Sciuridae: Tamias sibiricus / **Sister clade**	1.54	0.10
(16) Sciuridae: *Spermophilus francklini* / **Sister clade**	2.17	**0.05**
CASTORIMORPHA		
(17) Geomyidae: Castoridae / **Geomyioidea clade**	2.17	**0.05**
(18) Geomyidae: *Zyzogeomys* / ***Orthogeomys*** clade	3.04	**0.01**
(19) Geomyidae: *Chaetodipius fallax* / ***Chaetodipius penicillatus*clade**	2.27	0.07
MYOMORPHA		
(20) Anomaluromorpha / **Myomorpha**	1.81	0.07
(21) *Typhlomys* / **other Muroidea**	3.18	**0.02**
(22) Spalacidae / **Eumuroidea**	3.18	**0.02**
(23) *Calomyscus* / **other Muroidea**	1.97	0.06
(24) Nesomyidae / **other Muroidea**	1.97	0.06
(25) Cricetidae / **Muridae**	2.18	**0.05**
(26) Murinae: *Batomys* division / **other Murinae**	3.42	**0.05**
(27) Murinae : *Micromys* / **rest of*Rattus*clade**	4.08	**0.01**
(28) Murinae : *Chiropodomys* / **Sahul + Philippine murine sister-clade**	2.27	0.07
(29) Murinae :*Vandeularia* / **Sister clade**	4.03	**0.01**
(30) Murinae : *Golunda* / **Sister clade**	3.03	**0.02**
(31) Deomyinae / **Gerbillinae**	4.69	**0.01**
(32) Gerbillinae: *Taterillus* clade / ***Gerbillus*,*Meriones* clade**	1.66	0.09
(33) Gerbillinae: ***Gerbillus*clade**	3.38	**0.02**
(34) Deomyinae : *Deomys* / ***Acomys*,*Lophuromys* clade**	3.65	**0.01**
(35) Arvicolinae : **Arvicolinae** without *Prometheomys*	4.29	**0.01**
(36) Arvicolinae : *Dicrostonyx* clade / ***Microtus*clade**	2.07	**0.05**
(37) Arvicolinae : *Microtus xanthognathus* / **Sister clade**	2.23	0.06
(38) Neotominae : *Ochrotomys* / **Sister clade**	2.26	**0.05**
(39) Neotominae :*Baiomys*/*Scotinomys* clade / **Sister clade**	2.26	**0.05**
(40) Neotominae : *Peromyscus crinitus*clade / **Sister clade**	2.17	**0.05**
(41) Sigmodontinae : Sigmodontini tribe / **Oryzomyala**	1.55	0.10
(42) Sigmodontinae : *Oxymycterus* clade / ***Akodon*clade**	1.98	0.08
(43) Sigmodontinae : *Akodon cursor* clade / ***Akodon lutescens*clade**	2.61	**0.03**
(44) Sigmodontinae : *Aepeomys* / **Sister clade**	2.41	**0.04**
(45) Sigmodontinae : ***Thomasomys*clade**	3.05	**0.03**
(46) Sigmodontinae : *Eremoryzomys polius* / **Sister clade**	2.29	**0.05**
(47) Sigmodontinae : *Cerradomys*clade / **Sister clade**	2.29	**0.05**
(48) Sigmodontinae : *Sooretamys angouya* / **Sister clade**	2.56	**0.05**
(49) Sigmodontinae : Phyllotini and some Akodontini taxa / **Oryzomyini tribe sensu lato**	1.93	0.06
(50) Sigmodontinae *Wiedomys* / **Sister clade**	2.64	**0.04**
(51) Sigmodontinae : *Andinomys* / **Sister clade**	3.44	**0.02**
(52) Sigmodontinae : *Calomys* clade / ***Phyllotis* clade**	3.45	**0.02**

Clades can be found in the Additional file 2: Figure S2, Additional file 3: Figure S3, Additional file 4: Figure S4, Additional file 5: Figure S5, Additional file 6: Figure S6, Additional file 7: Figure S7, Additional file 8: Figure S8, Additional file 9: Figure S9, Additional file 10: Figure S10, Additional file 11: Figure S11, Additional file 12: Figure S12, and Additional file 13: Figure S13. _*Δ*1_represents the delta shift-statistics of [7] and [73].

Relationships significant at P = 0.05 are shown in bold. Clades in bold are the most diversified ones.

The gene supermatrix supports rodent monophyly (BP = 100%). Four main clades (Figure 1 and Additional file 1: Figure S1) are recovered : (1) the Ctenohystrica (BP = 100% , Additional file 2: Figure S2 and Additional file 3: Figure S3), (2) a squirrel-related clade (BP = 100%, Additional file 4: Figure S4 and Additional file 5: Figure S5), (3) the Castorimorpha (BP = 100%, Additional file 6: Figure S6) and (4) the Myodonta + Anomaluroidea (BP = 66%, Additional file 7: Figure S7, Additional file 8: Figure S8, Additional file 9: Figure S9, Additional file 10: Figure S10, Additional file 11: Figure S11, Additional file 12: Figure S12 and Additional file 13: Figure S13). The Myodonta + Anomaluroidea and the Castorimorpha clade are grouped in the mouse-related clade (BP =57%).

#### The Guinea-Pig related clade

Ctenohystrica (Additional file 2: Figure S2 and Additional file 3: Figure S3) is composed by the well-supported Ctenodactylidae + Diatomyidae (BP = 99%) and Hystricognathi ***sensu stricto*(BP = 81%)**[16]. Phiomorpha (Old World Hystricognathi) is paraphyletic (BP = 74%) with the Bathyergidae clade (mole rats, BP = 98%) being more closely related to Caviomorpha (South American Hystricognathi, BP = 100%) than to Hystricidae (Old World Porcupines, BP = 100%) [30]. Within the Caviomorpha, we recovered the dichotomy between Cavioidea + Erethizontoidea (BP = 84%) and Octodontoidea + Chinchilloidea (BP = 97%) [26]. The monophyly of these 4 superfamilies is also supported (BP > 95%). The intergeneric relationships within caviomorphs are in agreement with recent molecular phylogenies [35,39,44,54,71].

#### The squirrel-related clade

Within the squirrel-related lineage (Additional file 4: Figure S4 and Additional file 5: Figure S5), we recovered the reciprocal monophyly of Sciuridae (BP = 99%) and Gliridae (BP=100%). In agreement with the most recent phylogenetic analyses of rodents [28-30,35], our results corroborate the Aplodontidae + Sciuridae clade (BP = 100%). Within Gliridae, we found the same clades as those inferred by Montgelard *et al.*[37] and Nunome *et al.*[72]. Among Sciuridae, relationships are also well-resolved and the following lineages are recognized: the south-east Asian Callosciurinae (BP = 100%), the Xerinae (BP = 99%), and the Sciurinae (BP= 99%), that includes the Sciurini (BP = 100%) and Pteromyini (BP = 99%) tribes. These results are in agreement with previous hypotheses of Sciuridae composition and relationships [36,42].

#### The mouse-related clade

The monophyly of the mouse-related clade (Additional file 6: Figure S6 to Additional file 7: Figure S7, Additional file 8: Figure S8, Additional file 9: Figure S9, Additional file 10: Figure S10, Additional file 11: Figure S11, Additional file 12: Figure S12, Additional file 13: Figure S13) is poorly supported (BP = 57%). It contains three major clades: Anomaluroidea (BP = 100%), Castorimorpha (Castoridae + Geomyoidea ; BP = 100%) and Myodonta (BP = 100%) [21,27,29,30]. Myodonta is divided into Muroidea (BP = 100%) and Dipodidae (BP = 100%). Platacanthomyidae is the sister-group to all other Muroidea [49]. Our phylogeny corroborates the monophyly of Spalacidae (BP = 95%), Nesomyidae (BP = 100%), Cricetidae (BP = 99%) and Muridae (BP = 95%). Cricetidae subfamilies are also recovered as monophyletic in our analysis: Sigmodontinae (BP = 99%), Cricetinae (BP = 100%), Arvicolinae (BP = 99%), Tylomyinae (BP = 98%) and Neotominae (BP = 98%). Muridae as defined by recent analyses [40,41,74] is recovered monophyletic and includes the Acomyinae, Gerbillinae, Lophiomyinae and Murinae subfamilies. The monophyly of the Murinae (BP = 93%), Gerbillinae (BP = 100%) and Deomyinae (BP = 96%) subfamilies is also recovered [41,74]. Relationships and support within the muroids agree with those identified in the previous molecular phylogenies of [41,43], [46] and [47]. Our results suggested a sister clade relationship of Myodonta + Anomaluroidea with Castorimorpha. Castorimorpha (BP = 100%) is divided into the Castoridae (BP = 100%) and the Geomyoidea (BP = 99%). Our results also support the paraphyly of Heteromyidae with respect to the Geomyidae [28,30].

#### Impact of missing data

Molecular marker coverage is uneven among different taxa and between genomes. For example, sequencing effort for the Muridae has been very significant due to medical importance and genomic interests of model species (*cf.Mus musculusRattus norvegicus*). Furthermore, at the species level the mitochondrial genome has been better studied than the nuclear genome. Thus, mitochondrial genes have been sequenced for most of the available species within our dataset, and mitochondrial markers like *CYTB* (with 1152 sequenced taxa; Table 2) constitute the backbone of our phylogenetic inference. The single gene analysis of the *CYTB* provides a relatively similar topology at lower taxonomic (species level) but leads to either unresolved or conflicting results at higher taxonomic level (suborder, family, genus) compared to multigene topologies including nuclear genes, as attested by significant approximately unbiased (AU) [75] and Shimodaira and Hasegawa (SH) tests [76] (P < 0.05). By contrast, there have been relatively few nuclear gene studies addressing the phylogeny of lower level rodent relationships, except for some subfamilies, tribes and genera (*e.g.* Neotominae, Cricetinae, Oryzomyini, *MicrotusMusApodemusRattus*, and *Phyllotis*). At the species and subspecies level, Murinae is undersampled and only the higher-level taxonomic diversity (*i.e.* genus and family level) is represented by both nuclear and mitochondrial markers [41,43,46,47]. Capromyidae, Dipodidae, Gerbillinae, and African and Indonesian murines are understudied and not included in the present study (Table 3).

**Table 2:** Mitochondrial (mtDNA) and nuclear (nucDNA) loci used in this study

**Gene**	**Model**	**N taxa**	**N sites**
*12S rRNA* [mtDNA]	GTR+I+*Γ*	391	724
Breast and ovarian cancer susceptibility protein exon 11 (BRCA1) [nucDNA]	TVM+I+*Γ*	99	2977
Control region (*DLOOP*) [mtDNA]	HKY+I+*Γ*	45	996
Cytochrome oxydase 3 (*COX3*) [mtDNA]	GTR+I+*Γ*	105	784
Cytochrome b (*CYTB*) [mtDNA]	GTR+I+*Γ*	1152	1140
Interphotoreceptor retinoid-binding protein exon 1 (*RBP3*) [nucDNA]	GTR+I+*Γ*	536	1302
Growth hormone receptor (*GHR*) exon 10 [nucDNA]	HKY+I+*Γ*	282	974
*NADH* dehydrogenase 4 (*NADH4*) [mtDNA]	GTR+I+*Γ*	99	1389
*NADH* dehydrogenase 1 (*NADH1*) [mtDNA]	TVM+I+*Γ*	45	961
Recombination activating protein 1 exon 1 (*RAG1*) [nucDNA]	GTR+I+*Γ*	238	3044
von Willebrand gene (*vWF*) exon 28 [nucDNA]	TrN+I+*Γ*	110	1272

The abbreviated models are the following: HKY: Hasegawa, Kishino, Yano [79]; GTR: General Time Reversible [80,81]; TrN: Tamura-Nei [82]; TVM: Transversion Model; + *Γ*: variation in rates among sites modeled using a gamma distribution [83]; +I; a proportion of sites modeled as invariant [79]. N taxa = number of available taxa on public databases. N sites = Number of aligned nucleotides.

**Table 3:** Summary statistics for gene sequences available for rodent genera and species

**CLADE**	**Ngenera**	**gGENBANK**	**Percentage**	**Nspecies**	**spGENBANK**	**Percentage**
Rodentia	474	387	81	2261	1265	56
SCIUROIDEA	61	58	95,1	307	200	65
Aplodontidae	1	1	100	1	1	100
Gliridae	9	7	78	28	15	54
Sciuridae	51	50	98	278	184	66
CASTORIMORPHA	14	14	100	102	90	88
Castoridae	2	2	100	2	2	100
Geomyidae	6	6	100	40	33	83
Heteromyidae	6	6	100	60	55	91
CTENOHYSTRICA	72	59	82	275	158	57
Abrocomidae	2	1	50	10	2	20
Bathyergidae	5	5	100	15	14	77
Capromyidae	6	1	17	8	1	7
Caviidae	6	6	100	18	13	89
Chinchillidae	3	3	100	7	6	86
Ctenodactylidae	4	2	50	5	3	60
Ctenomyidae	1	1	100	60	38	65
Cuniculidae	1	1	100	2	2	100
Dasyproctidae	2	2	100	13	5	38
Diatomyidae	1	1	100	1	1	100
Dinomyidae	1	1	100	1	1	100
Echimyidae	21	17	81	86	44	54
Erethizontidae	5	4	80	17	7	44
Hystricidae	3	3	100	11	7	64
Myocastoridae	1	1	100	1	1	100
Octodontidae	8	8	100	13	11	85
Petromuridae	1	1	100	1	1	100
Thryonomyidae	1	1	100	2	1	50
ANOMALUROMORPHA	4	3	75	9	4	44
Pedetidae	1	1	100	2	1	50
Anomaluridae	3	2	67	7	2	29
MYOMORPHA	324	253	78	1568	813	52
Dipodidae	16	7	44	51	10	20
MUROIDEA	308	246	79	1516	803	53
Platacanthomyidae	2	1	50	2	1	50
Arvicolinae	28	25	89	151	111	74
Calomyscidae	1	1	100	8	2	25
Cricetinae	7	6	86	18	14	78
Deomyinae	4	4	100	42	33	48
Gerbillinae	16	14	88	103	40	39
Leimacomyinae	1	0	0	1	0	0
Lophiomyinae	1	1	100	1	1	100
Murinae	124	88	70	560	231	41
Neotominae	16	16	100	124	103	83
Nesomyidae	21	17	81	61	28	43
Otomyinae	3	3	100	23	16	52
Sigmodontinae	74	61	82	377	231	61
Spalacidae	6	6	100	36	9	25
Tylomyinae	4	3	75	10	3	30

Ngenera and Nspecies represent the number of genera and species described in Wilson and Reeder (2005); gGENBANK and spGENBANK represent the number of genera and species available in GENBANK; Percentage represents the proportion of genera and species included in our study.

The present phylogeny is the most comprehensive hypothesis for rodent species and generic relationships up to date and provide substantial improvement in comparison with previous studies (Bininda-Emonds *et al*[61]). Despite the 75% of missing data, the ML trees (summarized in Additional file 1: Figure S1 and Additional file 2: Figure S2, Additional file 3: Figure S3, Additional file 4: Figure S4, Additional file 5: Figure S5, Additional file 6: Figure S6, Additional file 7: Figure S7, Additional file 8: Figure S8, Additional file 9: Figure S9, Additional file 10: Figure S10, Additional file 11: Figure S11, Additional file 12: Figure S12, and Additional file 13: Figure S13) corroborate recent findings [16,29,30,35,36,38,40-43,47,77,78] with bootstrap values (BP) > 70% for 64% of the nodes. This suggests that despite a large proportion of missing data the present molecular character sample provides information about rodent evolutionary affinities. Simulations and large scale analyses have shown that missing data may not lead to inaccuracies in phylogeny reconstruction. As an example, Wiens [84] concluded that “the reduced accuracy associated with including incomplete taxa is caused by these taxa bearing too few complete characters rather than too many missing data cells”. Philippe *et al*[85] came to the same conclusion using a eukaryote protein supermatrix and computational simulations, and remarked that as much as 75% of the data could be missing without significantly decreasing the reliability of the phylogeny produced. AU [75] and SH tests [76] were used to compare our best topology with trees inferred from two reduced datasets containing 56% (*i.e.*, 1254 taxa and 4130 sites) and 39% (*i.e.*, 371 taxa and 4130 sites) of missing data respectively. Topological tests did not find significant difference (P > 0.05) between the best tree and the topological hypothesis obtained from both reduced datasets. Our findings corroborate results of [84] and [85] as we recovered most relationships inferred in previous works at lower taxonomic levels, an indication that enough informative characters were present to mitigate the effect of missing data. Of course, we acknowledge that the rodent phylogeny here presented has to be ameliorated because of the suboptimal gene and taxon coverage, but we really think it is a reasonable approximation of the rodent phylogeny which accuracy is sufficient to allow for diversification analyses.

### Imbalance and shifts in diversification rate within rodents

**Figure 3:**
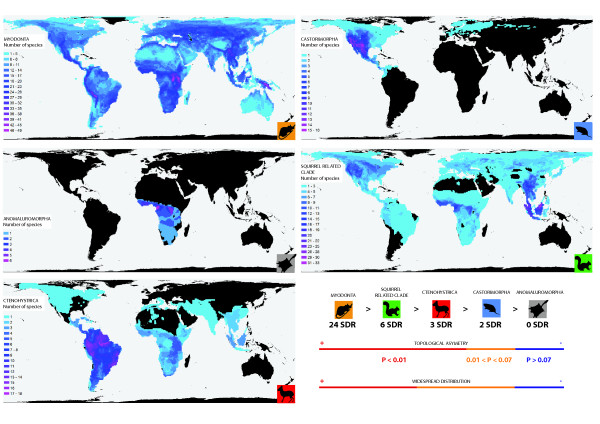
**Current distribution maps of the major rodent clades.** Color gradient represents species richness - a warmer color indicates higher richness. Black corresponds to areas where the group is not present. The maximum number of species in a cell (warmest color) for each clade is 49 (Myodonta), 16 (Castorimorpha), 6 (Anomaluromorpha), 33 (squirrel-related clade), and 18 (Ctenohystrica) respectively. Distribution width and topological asymmetry are indicated on the lower right part of the figure, together with the number of significant SDR (shifts in diversification rate).

Whole-tree tests conducted on the complete species sampling indicate significant variation in diversification rates among rodent lineages (Table 4, Figure 3). Except for Castorimorpha and Anomaluromorpha, all P-values of the 4 topology-based indices of whole-tree symmetry (IC, MΠ*, Mσ*, B1) within rodent subclades (Myomorpha, Sciuroidea, Ctenohystrica) ranged from significant (P <0.05) to highly significant (P <0.001). Myomorpha is the most imbalanced clade followed by Sciuroidea, Ctenohystrica, Castorimorpha and Anomaluromorpha (Table 4). The significant shifts in diversification rate (at P < 0.05 level) within rodent taxa under the delta shift statistics (_*Δ*1_) are reported in the Table 1, Figure 2 and Additional file 1: Figure S1, Additional file 2: Figure S2, Additional file 3: Figure S3, Additional file 4: Figure S4, Additional file 5: Figure S5, Additional file 6: Figure S6, Additional file 7: Figure S7, Additional file 8: Figure S8, Additional file 9: Figure S9, Additional file 10: Figure S10, Additional file 11: Figure S11, Additional file 12: Figure S12, and Additional file 13: Figure S13. The (*Δ*_1_) statistics suggests that unequivocal shifts in diversification rate (SDR) occurred within the 4 major rodent clades, with two significant SDR (0.01 < P < 0.05) in Castorimorpha, 3 significant SDR in Ctenohystrica, 6 significant SDR within the squirrel-related clade, and 24 significant SDR in Myomorpha. We also detected one, 4, 3, and 9 marginally significant SDR (0.05 < P < 0.10) in Castorimorpha, Ctenohystrica, the squirrel-related clade and in Myomorpha respectively.

**Table 4:** Tests of among-clade diversification rate using 4 topology-based indices of whole-tree symmetry in Rodentia

**Clade**	**IC**	**M*Π***	**M*σ***	**B1**
	**Min (0.025)**	**Min (0.025)**	**Min (0.025)**	**Min (0.025)**
	**Max (0.975)**	**Max (0.975)**	**Max (0.975)**	**Max (0.975)**
Rodentia	35928−35412	(−)0.912(−)0.905	0.574−0.575	1087.040−1084.840
	0.001−0.001	0.001−0.001	0.001−0.001	0.001−0.001
Myomorpha	23741−23739	(−)0.911(−)0.904	0.578−0.571	761.502−760.302
	0.001−0.001	0.001−0.001	0.001−0.001	0.001−0.001
Sciuridae	2778−2655	(−)0.813(−)0.754	0.607−0.628	150.188−156.420
	0.001−0.001	0.001−0.001	0.001−0.001	0.001−0.006
Ctenohystrica	2233−2093	(−)0.707−(−)0.630	0.628−0.660	136.493−140.953
	0.001−0.001	0.001−0.001	0.001−0.002	0.001−0.010
Castorimorpha	549−507	(−)0.705−(−)0.618	0.620−0.669	49.591−52.810
	0.020−0.040	0.004−0.030	0.002−0.060	0.004−0.170
Anomaluromopha	5−0	(−)0.080−(−)0.802	0.901−1.100	3.302−4.000
	0.800−1.000	0.800−0.001	0.600−0.800	0.620−1.000

Values represent the test statistics with the P-value on the second line for each clade. The range of values represents the upper and lower bounds generated when the analyses were repeated with 1,000,000 random resolutions of polytomies with different degrees of symmetry. Indices are Colless Index (IC), Shao and Sokal Index (B1) and the M statistics M*Π*and M*σ*.

Diversification rate varies significantly in rodents, a pattern also observed in bats and lagomorphs ([86], Figure 3 and Table 4) and other vertebrate clades [87,88]. Within Rodentia, Myomorpha displayed higher imbalance in comparison to Sciuroidea, Caviomorpha, Castorimorpha and Anomaluromorpha. Our results suggest that rodents underwent a number of significant SDR, especially within the Cricetidae, Muridae, Sciuridae and Octodontoidae. These different imbalance signatures suggest distinct diversification processes among rodent lineages. Many hypotheses have been proposed to explain these evolutionary radiations. The most common explanations are key innovations (***e.g.*** hypsodonty or teeth patterns like the murine or cricetine dental plans, [40]), events related to biogeographical history (*e.g.* colonization of south America by Sigmodontinae; [41,89]), extinction of competitors (e.g. multituberculate and plesiadapid extinction through the Paleogene; [90]), a predator absence (*e.g.* insular New Guinea / Papua murine diversification), and / or environnemental changes (*e.g.* opening of habitats). All these factors could have played a role during the rodent radiations.

The most imbalanced clade is the Myodonta (Muroidea + Dipodidae). Most shifts in diversification rates (Table 1, Figure 2) are located within the two most speciose muroid families: the Cricetidae (681 species) and the Muridae (728 species). Of the 24 significant SDR, 7 and 14 shifts are located within Cricetidae and Muridae respectively. For Cricetidae, accelerations of the diversification rates were found for 3 clades of Neotominae (North-American Cricetidae), 10 for Sigmodontinae (South-American Cricetidae) and two within Arvicolinae (voles). Within the Muridae, accelerations in the diversification rates are found for one clade of Deomyinae, two of Gerbillinae and 4 within Murinae. The outstanding muroid diversity in both tropical and boreal habitats is peculiar within the evolutionary history of the placentals (Figure 3). Muroid rodents comprise 28% of mammal species and this superfamily [13] is larger than any other non-rodent orders. Our analyses agree with three conclusions of Steppan *et al.*[41], who delineated 4 bursts of speciation within their Muroidea timetree: *(i)* the initial radiation of the Eumuroidea (SDR 22), *(ii)* the radiation among cricetid families (SDR 25), *(iii)* the initial radiation among Oryzomyala sigmodontines (SDR 41) [89,91], and *(iv)* the initial radiation among the Murinae (at the exception of *Batomys* division (SDR 26)). To explain these speciation bursts, they referred to an increase in speciation rate due to evolutionary and biogeographic events. In fact, the major centers of the muroid diversification overlapped most continents in both hemispheres: America and Palearctic for Cricetidae, and Old World and Sahul for Muridae. Key opportunities, such as colonization of new areas are well-known for driving speciation and have contributed significantly to the diversification of organisms [92,93]. These dispersal events led some organisms to exceptional evolutionary [94-97] or adaptive radiations [2,98-100]. The importance of colonization is considered essential for the radiations and acceleration of the speciation rates within Muroidea. Some works on murinae have recently confirmed the role of biogeographic events in driving shifts in diversification, for example the colonization of the Sahul by Murinae [47] (SDR 28), the radiation of *Rattus* in South East Asia and Sahul [51] (SDR 27) and the colonization of Africa by the *PraomysMus* and *Arvicanthis* lineages (SDR 29) [46,101]. The radiation of Sigmodontinae have also been related to the colonization of the South American continent [91]. From the beginning of the Miocene (24.7 +/- 1.1 Mya *cf.*[41]), the radiation of Muroidea have caused a major turnover in the composition of rodent lineages as suggested by the fossil record [102,103] and by our topological-shape results. Our study (Figure 3), as well as recent phylogenetic works on Eumuroidea clades strongly support the role of colonization processes in SDR. In addition, lineages that originated during these radiations exhibited a broad array of both ecological generalist and specialists within different colonized areas. Compared to the other rodent clades, Muroidea includes smaller sized and less “specialized” taxa [104]. The high diversity pattern in such small size taxa has been linked to the shortest generation time among terrestrial mammals [105] and to a better partition of ecological niches [106]. Evidence so far is consistent with these hypotheses, for instance previous works on primates and carnivores have found marginally significant association between diversification and body mass [11,12]. Muroids displays the highest molar diversity among Rodentia associated to a wide number of dental vicariants due to convergent evolution [40,107-109]. Their small size, their teeth diversity and their “generalist” morphology could be linked to their recent success. They have succeeded to colonize new areas and to diversify in more habitats than their more specialized sister clades (*e.g.*, arboreal squirrels, porcupines, mole rats, and ancient American endemics like Caviomorpha and Castorimorpha).

Within Sciuridae, two significant SDR (P < 0.05) occurred at the origin of the family, and along the branch leading to the Sciurillini tribe and the rest of the Sciuridae, two within the Callosciurinae subfamily, one within the Pteromyini tribe, and then one significant SDR within the Xerinae subfamily. The Sciuridae is characterized by a wide geographic distribution (Figure 3) and a high specific diversity (278 species) associated to many adaptive trends (terrestrial, arboreal and gliding). Mercer and Roth [36] showed that Cenozoic global changes mediated their diversification history. After the Eocene, the colonization of major land masses by the Sciuridae have led to their diversification within forest or open habitats. The squirrel-related clade is widespread like Myomorpha but compared to Muroidea its members display more constrained adaptations and morphologies. These differences could explain why they have higher imbalance index (Table 4, Figure 3) than Ctenohystrica and Castorimorpha and less than Myomorpha. Most of the Ctenohystrica radiation is represented by Caviomorpha which have undergone endemic evolution in South America. Caviomorpha have colonized South America from Africa [35,110,111]. We did not detect a significant shift at the root of Caviomorpha in our analysis despite their high diversity. Such a result could be a consequence of the extinction of taxa of the earliest Caviomorpha radiations [112-115]. Octodontoidea are the most speciose suborder within the Caviomorpha comprising the Echimyidae (South American spiny rats), the Ctenomyidae (tuco-tucos), the Abrocomidae and the Octodontidae. They underwent an adaptive radiation in South America during the Miocene with scansorial (*Capromys*), fossorial (Ctenomyidae), terrestrial (*Trinomys*), semi aquatic (*Myocastor*) and arboreal (*Echimys*) representatives. Concerning Echimyidae, Galewski *et al.*[44] did not resolve the origin of this clade with one nuclear gene, a pattern possibly associated with rapid diversification events. They invoked the role of paleoclimatic variation as a driving force through their radiation in the Miocene. Our results converge on the same conclusion with two shifts occurring at (1) the split between Caviidae *vs.* Dasyproctidae (SDR 2) and (2) the divergence between Echimyidae and (Ctenomyidae+Octodontidae) (SDR 3). These clades display adaptations to open habitats (Caviidae and (Ctenomyidae + Octodontidae)) or forest habitats (Dasyproctidae and Echimyidae) where they subsequently diversified. Miocene climatic changes in South America may have played a major role in the diversification of Caviomorpha as suggested by the fossil record [116,117], molecular dating results (herein and also [26,44,54,111]) and our SymmeTREE results. Castorimorpha and Anomaluromorpha clades display high morphological and ecological constraints with fossorial (Geomyioidea), gliding (Anomaluridae) or jumping (Pedetidae and Heteromyidae) adaptations. Moreover they display high endemism like Caviomorpha (Figure 3). Anomaluromorpha are only found in Africa and Castorimorpha are mainly distributed in North America (except *Castor fiber*) (Figure 3). Their geographical distribution and their specialized morphology could explained the difference in the imbalance analyses and the low number of inferred SDR in comparison to other rodent clades.

Investigating correlates of diversification shifts for the Rodentia remains a challenge, and a variation in a single trait is unlikely to explain all shifts detected. In this framework, methods incorporating paleoclimatic and biogeographic information would be informative. Such an approach could be useful for clades such as the Cricetidae or Muridae where numerous shifts in diversification were recorded.

### The Paleogene / Neogene contrast of the rodent timetree

Calibrating phylogenetic trees is a difficult problem for data with a patchy taxonomic sampling and markers with heterogeneous patterns of molecular evolution. Likelihood ratio tests [118] rejected the molecular clock for the 11 genes. This result is not surprising as rates variations have been evidenced for rodent mitochondrial and nuclear genes. To get maximum dating signal, genes were analyzed in combination to infer divergence times. Calibration of our ML trees using the partitioned Bayesian relaxed clock model of [119,120] provides an estimate of the rodent timetree (Figure 1). All analyses with different MCMC sampling converged to the same divergence time estimates.

Our supermatrix-based molecular clock approach simultaneously calibrated by multiple fossil constraints provides an alternative to previous dated supertrees [61] because we use the concatenated information of independent molecular markers rather than averaging over independent source analyses.

Molecular dating here suggests that many extant families originated during the Paleogene. The divergence dates of rodent families indicate that they were all established before the end of Oligocene (Median family age= 31 Mya). The majority of radiations leading to extant rodent diversity seems to have occurred during the Neogene (Median age of generic radiation = 22 Mya) with some exceptions such as the older diversification of the Sciuroidea or the Phiomorpha families. Analysis of diversification rates shows that statistically significant (P < 0.05) and substantial diversification shifts (0.05 < P < 0.1) were concentrated in the Neogene, and that the majority of SDR occurred around 10 Mya during the middle Miocene. Means of the absolute value of the delta shift statistic for nodes of the rodent clades in each geological epoch are presented in the Figure 2 (Upper Part: B). We obtained the largest values from Paleocene intervals (65.5-55.8 Mya) (Figure 2 B). Mean values in the SDR are significantly different among time intervals (one-way ANOVA, F 5,1259 = 13.42, P < 0.01). The mean value for the Paleocene (65.5-55.8 Mya) is significantly larger than in the Pliocene and Quaternary (Tukey test P < 0.01 and P < 0.01), and is not significantly different from the Eocene, Oligocene and Miocene time intervals (Tukey test P < 0.40, P < 0.07, P < 0.09). We examined the distribution of species in each clades that were present since 65.5 Mya to identify which lineages were responsible for the large SDR within different geological periods. The lineages leading to Myodonta, Sciuroidea+Gliridae, Castorimorpha and Ctenohystrica were present before 65.5 Mya and displays most of the extant diversity of rodents. During the 60-40 Mya period, the first rodent families emerged in the fossil record and explosive radiations took place [121]. Because there is no significant difference in SDR from the Paleocene to the Miocene, it seems that rodent clades have diversified at a fairly constant rate during these epochs.

Rodents have undergone regular Shifts in Diversification Rate (SDR) through the Cenozoic (Figure 2). Among the 35 significant SDR (see previous section), only six took place during the Paleogene. However, the fossil record has revealed that the Paleogene was a period of intensive rodent diversification with the appearance of 9 new families (*i.e.* Cylindrodontidae, Eutypomyidae, Sciuravidae, Gliridae, Zegdoumyidae, Chapattimyidae, Cocomyidae, Ivanantomyidae, and Yuomyidea) [122]. According to their period of diversification (*i.e.* Paleogene or Neogene), two groups emerged from the timeline analysis: the first included the sciurid-related clade and the Castorimorpha, whereas the second included Myodonta, Anomaluromorpha and Ctenohystrica. The first group is characterized by older generic divergences and a higher density SDR through the Paleogene and this is also attested by the richness and occurence of the fossil record of Gliroidea [123-125], Aplodontoidea [126,127] and Castorimorpha [102]. In the second group, the mouse-related clade and Ctenohystrica have the majority of generic divergences and SDR through the Neogene. Within Muroidea, even if stem Cricetidae occurred in the Eocene and Oligocene fossil records [128], it is now clear that the extant subfamilies diversified during the Neogene. Numerous cladogenesis events are identified during the Neogene within the Muroidea, especially in the Cricetidae and Muridae (Figure 1 and 2) that represent the most important and recent evolutionary radiations. This result is congruent with the richness of their fossil record during the Neogene (Figure 2 - [103,128-130]).

Comparisons between results from our diversification analyses and the available fossil record point to a late Paleogene or Neogene radiation of extant rodent lineages. The extinction of stem lineages could also explain the low number of speciation events detected in most stem branches. These results corroborate the macroevolutionary study of Bininda-Emonds *et al *[61] who observed a delay between the KT boundary and the Neogene regarding the diversification of placentals (see also [62,131]). The long branches leading to Geomyoidea or extant Ctenodactyloidea (Ctenodactylidae + Diatomyidae) could be explained by the extinction of stem Castorimorpha and Ctenodactyloidea. The diversification of crown rodents from the late Eocene onwards coincides with the extinction or decline of the major Paleogene fossil groups (Figure 2C - [103]). Several extinct groups, without extant relatives (*e.g.* Theridomorpha, Ischyromyoidea, Ctenodactyloidea, and Sciuravida), disappeared or declined in the Oligocene and the Neogene (Figure 2C). Simultaneously, most of the relatives of extant species played a major role in rodent communities during that period, in particular the Muridae and Cricetidae (Figure 2 and 3). Because extinction processes may have biased the interpretation of SDR, future studies should incorporate fossil data in supermatrix/supertree inferences.

## Conclusions and Perpectives

The present study is a first attempt to provide a phylogenetic synthesis to be used for comparative meta-analyses of rodent evolution (topology are available in the Additional file 14). We demonstrated that the diversification rates of rodent taxa were not constant through time and some clades have experienced significant shifts in diversification rates. Our results show that most widespread and diversified clades (Myodonta and the squirrel-related clade) display a higher degree of topological asymmetry and more SDR. Recent opportunities to colonize new geographical areas must have driven speciation and contributed significantly to the diversification of both groups. Numerous SDR are evidenced through the Tertiary, but at different periods for each clade. The majority of these shifts occurred for the most recent familial rodent radiations: the Cricetidae and Muridae clades. Comparison between the rodent fossil record and our results suggest that extinctions led to the loss of diversification signal for the Paleogene nodes. The main perspective of this study is to provide a framework for comparative studies of rodents and an update of large scale phylogenies of this order. The ML trees (summarized in Additional file 1: Figure S1 and Additional file 2: Figure S2, Additional file 3: Figure S3, Additional file 4: Figure S4, Additional file 5: Figure S5, Additional file 6: Figure S6, Additional file 7: Figure S7, Additional file 8: Figure S8, Additional file 9: Figure S9, Additional file 10: Figure S10, Additional file 11: Figure S11, Additional file 12: Figure S12, and Additional file 13: Figure S13) corroborate recent multigene analysis with bootstrap values (BP) > 70% for 64% of the nodes. The occurence of taxa not studied in a phylogenetic framework and lack of DNA data for many of the genetic markers, however, constitute the main challenge for the further clarification of rodent evolution.

One avenue for further research is to explore the morphological / biogeographical drivers of diversification. The use of ancestral character reconstruction methods will be required to test if there are correlations between phenotypic innovations or biogeographic events and diversification in rodents. The exploration of macroevolutionary patterns and their link with morphological innovations, biogeography or climatic events is a key for a better understanding of the mammalian Cenozoic radiations.

## Methods

### Taxonomy

All species names followed the rodents classification of Carleton and Musser [132]. We chose this classification – recognizing about 2,261 rodent species – because it is the most recent update, and it is widely used and cited in the mammalian biology literature. We added the newly discovered genus *Laonastes *[14,16] which had not been described in reference [132]. The Carleton and Musser [132] taxonomy provides the most recent and accepted species list for Rodentia that also includes species synonyms. Tracing synonyms is essential for establishing congruence among different gene datasets that have used different names for the same taxa. Synonyms that coud not be traced in public databases for available molecular markers were excluded from subsequent analyses.

### Sequence data

In order to collect suitable candidate genes for the supermatrix assembly, DNA sequences of rodents were downloaded from EMBL / GenBank / DDBJ databases. Keyword frequency searches were performed to collect genes that were sequenced over a large taxonomic range using rodent species and genus names [132]. For these searches we focused on genes that have been previously used to infer rodent phylogenies. Refined searches were then performed using the rodent section of the NCBI taxonomic browser and BLAST [133] searches on euarchontan assembled genomes (mouse, rat, rabbit, human and rhesus macaque). This cross-search allowed for the retrieval of an extensive dataset of all rodents DNA sequence data available in public repositories. If multiple DNA sequences were available for the same taxon we checked its monophyly by using literrature and keep the most complete of the fragments prior to subsequent analyses. During the course of our study some additional sequences become available (e.g. [53,134]) but were not included in the analyses.

In this study we focused on the 11 nuclear and mitochondrial markers which allow us to maximise rodent species sampling (Table 2 and Table 3). Following this procedure we harvested 1,265 DNA sequences. The resulting dataset represents 100% of the families (33 families), 81% of the genera (387 of 474 genera), and 56% of the species (1,265 of 2,261 species) of rodents currently recognized in Wilson and Reeder [132] and recent phylogenetic works were also taken into account (eg. [50,89,135-141]). The rodent taxonomy adopted for the present study followed references [25,27,132] and is provided as Additional file 15. Due to the size of this dataset many taxa suffer from large amount of missing data, but all share at least one mitochondrial or nuclear gene, thus avoiding the problem of non overlapping sequences [142].

The rodent outgroups were chosen among the Euarchontoglires [143] for which genomes were available (*OryctolagusMacacaHomo*). If available, one Scandentia (*Tupaia*), one Dermoptera (*Cynocephalus*) and two additional Lagomorpha (*OchotonaLepus*) outgroups were added to each gene. DNA sequences were aligned with MUSCLE [144] and subsequently checked by eye with SEAVIEW [145]. For the 12S rRNA and 16S rRNA alignments, ambiguous positions were eliminated using the Gblocks program (version 0.91b, [146]) with the following options: a minimum of half the number of sequences for a conserved position and for a flank position, a maximum of 8 contiguous non-conserved positions, a minimum of 2 sites for the block length after gap cleaning, and all gap positions can be selected. The supermatrix concatenate contains 1265 rodent taxon sequences aligned for 15,535 sites, with 75% of missing character states. If necessary, non overlapping sequences (e.g. sequences available for different species of the same genus) were eliminated from the matrix. All genes are described in Table 2 and all datasets are available online (also see additional file 16 and Additional file 17 for accession numbers).

### Phylogenetic analyses

The general time reversible (GTR) model plus invariable sites and Gamma (*Γ*) distribution [81] was selected as the best fit under the AIC criterion using Modeltest 3.04 [147]. The dataset was partitioned by codon positions for exons. Maximum likelihood (ML) analyses were run with RaXML version 7.0.4 [148]. For the dataset partitioned only by gene and codons, we applied to each partition the GTRGAMMA (GTR+*Γ*) + Invariant site option. For the gene-codon-partition dataset, we used the GTRMIX option of RAxML. The GTRMIX option assumes the faster GTRCAT model for the topological search, but then uses the GTRGAMMA model when computing the likelihood value of the topology. Each RAxML run comprised 100 tree search replicates (with the default parameters).

Node support for codon/gene-partioned datasets was estimated by the means of non-parametric bootstrap resampling [149]. Bootstrap proportions (BP) were calculated with the following procedure: 100 pseudoreplicates for the supermatrix and 1000 pseudoreplicates for each single-gene matrix. Pseudoreplicate trees were inferred using the ML method in RAxML under a GTRMIX model.

In order to evaluate the impact of missing data on our inference we built two additional matrices: *(1)* a supermatrix containing the 4 genes with best taxonomic coverage (cf. *12S rRNA* + *CYTB* + *RBP3* + *GHR* ; 56% of missing character states) and *(2)* a supermatrix containing the same 4 genes (39% of missing data) but maximizing the taxon sampling at the genus level. These supermatrices were subsequently analysed with RaxML following the same procedure as described for the 11-gene supermatrix. The two inferred topologies were compared to the 11-gene topology after restriction to the subset of shared taxa and using the approximately unbiased (AU) [75] test as implemented in CONSEL [150]. PAUP* version 4.0b10 [151] was used to calculate the site likelihoods for each of the test topologies with the GTR + I + *Γ* model as specified using the output from Modeltest 2.2. The CONSEL analyses employed 10 batches of 10^6^ bootstrap replicates.

### Diversification rate analysis

To estimate diversification rates we used the phylogeny with the complete taxon sampling according to the classification of reference [132]. Species for which no DNA data were available were grafted to the most recent common ancestor of the closest relative taxon available within our molecular framework, *i.e.,* a species of the same genus, or tribe, or family. In this way, one composite topology was generated from the supermatrix analyses and the taxonomic list.

To study species diversification patterns, 4 topology-based indices of whole-tree symmetry were employed [7,152,153]. All 4 methods (IC, MΠ*, Mσ*, B1) use an equal rates Markov (ERM) model of clade growth [154] to test how well a tree fits to the equal-rates null hypothesis. A taxonomic imbalance in extant lineages is found if nonrandom diversification has taken place. Each topological-based statistic was calculated using a Monte Carlo simulation of its null distribution using 1,000,000 tree topologies of the same size as our rodent phylogeny, but generated under an ERM model. We used this approach on the complete topology. Analyses of tree symmetry and identification of diversifying clades were performed with SymmeTREE version 1.1 [7,73]. Because polytomies in the tree may bias SymmeTREE analysis [7], they were treated as soft.

To identify the nodes of the tree that show significant imbalance, the delta-shift method (_*Δ*1_) was used [7]. This likelihood topological-based method searches for significant shifts in diversification rates (SDR), and incorporates information on the distribution of taxonomic diversity over the entire tree. The delta shift-statistics determines the diversification rate shift probability along the internal branch of a local triplet tree that includes the two basal-most ingroup clades and a local outgroup. The three-taxon computations are replicated over all internal branches to check for diversification rate shifts within the whole tree [7]. The _*Δ*1_distribution was obtained by means of Monte Carlo simulation of its null distribution, using 1,000,000 topologies of the same size as the final tree, but generated under an ERM model.

### Estimating divergence times within rodents

Ideally, all the 1,265 species would have been analysed simultaneously within a single molecular dating analysis. However probabilistic search algorithms become prohibitively slow for a large number of taxa and are less likely to identify an optimal dated topology. In an attempt to approach this problem and to reduce computational time, a compartimentalization approach [155] was used. The global chronogram was constructed after analysis of hierarchically nested supermatrices. Ultimately, 8 supermatrices (Sciuroidea; Ctenohystrica; Castorimorpha; Anomaluromorpha + Dipodoidea + Platacanthomyidae + Spalacidae; Sigmodontinae + Tylomyinae; Neotominae; Arvicolinae + Cricetidae; Murinae; Gerbillinae + Acomyinae + Lophiomyinae) were built with subsamples of genes as indicated in Additional file 18. We used BEAST v1.6 [119,120] to estimate the divergence dates within our 8 supermatrices, by applying the best fitting model, as estimated by MODELTEST 2.0 to each of the partitions. We assumed prior Yule speciation process and an uncorrelated lognormal distribution for the molecular clock model [156]. Default prior distributions were used for all other parameters, and two independant MCMC chains were ran for 200 million generations. The program Tracer [157] was used to assess convergence diagnostics, and showed that each run reached similar date estimates for all nodes.

Three calibration constraints based on paleontological estimates and previously used for rodent molecular dating studies [16,41,71] were incorporated: the Caviomorpha radiation (28.5 to 37 Mya ; [158,159]), the Aplodontidae / Sciuridae divergence (37 to 50 ; Myr, [103]), and the *Glis* - *Dryomys* split (28.5 to 50 Mya ; [122]). Based on the 61.5 to 100.5 Mya estimate for the divergence between Lagomorpha and Rodentia [160], the a priori expected root height was set to 100 Mya with a standard deviation of 50 Myr. Finally, the overall dated tree was reconstructed by combining results from the hierarchically nested supermatrices.

The resulting chronogram has been used to study the occurrences of significant SDR throughout the Tertiary. To do so we followed the methodology of Jones *et al.* (2005), using speciation date estimates to calculate the mean of the absolute value of the delta shift statistic (_*Δ*1_) in each geological epoch (1 : Paleocene (65.5-55.8 Mya), 2 : Eocene (55.8-33.9 Mya), 3 : Oligocene (33.9-23.03 Mya), 4 : Miocene (23.0-5.3 Mya), 5 : Pliocene (5.3-1.8 Mya), 6 : Quaternary (1.8-0 Mya)).

### Clades distributions and richness

Clades distributions / species richness maps were created using gridded species distribution data from Fritz and collaborators [161,162]. Grid cells with equal surface of 9309.6 square kilometers were used. Species presence/absence was recorded for each species and each cell for all the species in every major lineage. Species richness was then calculated as the total number of species co-occurring in every cell. The overlap of the species distributions is used to represent the distribution of the higher level taxon to which they belong and the color gradient within its range represents species richness. Areas where the lineage is not present are left black. Resulting maps were drawn using the Behrmann projection and manipulated in ArcGIS 9.3 computer program (ESRI Inc.).

## Authors contributions

P-HF designed the project, planned and conducted analysis and wrote the manuscript. All authors analysed data and contributed to the writing and editing of the manuscript. All authors approved the final manuscript.

## Supplementary Material

Additional file 1 **Figure S1.** Rodent species level evolutionary tree. Species-level phylogenetic topology based on the highest-likelihood tree inferred from the 11-gene supermatrix, and combined with the taxonomic information of Wilson and Reeder (2005). Click here for file

Additional file 2 **Figure S2.** Cladogram depicting the highest-likelihood topology for the Ctenohystrica. Circles at nodes represent bootstrap support (circles: black 100-95%, white 95-70%, gray 70-50%). Maximum likekihood tree (lnL=-714749.2). Displayed clade are highlighted using the simplified full ML topology on the left side of the figure. Molecular marker sampling is depicted for each taxa to the right of the tree. Names of the genes are given. Genes sampled for our dataset (Table 2) are marked in black square while missing genes are symbolized by white squares. Triangles indicate significant (P < 0.05) and marginal (0.05 < P < 0.1) shifts in diversification rate (SDR) as inferred by _*Δ*1_. LA = Diatomyidae, CT = Ctenodactylidae, TH = Thryonomyidae, PE = Petromuridae, CU = Cuniculidae, DASYPROC = Dasyproctidae, DI = Dinomyidae, CHINCHILLID = Chinchillidae. Bootstrap for all nodes and topology can be found in Additional file 14. Click here for file

Additional file 3 **Figure S3.** Cladogram depicting the highest-likelihood topology for the Octodontoidea. See Additional file 2: Figure S2 for details of the legend. AB = Abrocomidae, CA = Capromyidae. Click here for file

Additional file 4 **Figure S4.** Cladogram depicting the highest-likelihood topology for the Sciuroidea. See Additional file 2: Figure S2 for details of the legend. AP = Aplodontidae, RA = Ratufinae, SI = Sciurillinae. Click here for file

Additional file 5 **Figure S5.** Cladogram depicting the highest-likelihood topology for the Xerinae. See Additional file 2: Figure S2 for details of the legend. Click here for file

Additional file 6 **Figure S6.** Cladogram depicting the highest-likelihood topology for the Castorimorpha. See Additional file 2: Figure S2 for details of the legend. CAS = Castoridae. Click here for file

Additional file 7 **Figure S7.** Cladogram depicting the highest-likelihood topology for the mouse-related clade. See Additional file 2: Figure S2 for details of the legend. PED = Pedetidae, ANO = Anomaluroidea, PLA = Platacanthomyidae, Rhy = Rhyzomyidae, Spalac = Spalacidae, CAL = Calomyscidae, LOP = Lophiomyinae. Click here for file

Additional file 8 **Figure S8.** Cladogram depicting the highest-likelihood topology for Sigmodontinae [part 1] + Tylomyinae. See Additional file 2: Figure S2 for details of the legend. Tyl = Tylomyinae, Ich = Ichthyomyini, Rei = Reithrodontini. Click here for file

Additional file 9 **Figure S9.** Cladogram depicting the highest-likelihood topology for Sigmodontinae [part 2]. See Additional file 2: Figure S2 for details of the legend. Tyl = Tylomyinae, Ich = Ichthyomyini, Rei = Reithrodontini, Phy = Phyllotini. Click here for file

Additional file 10 **Figure S10.** Cladogram depicting the highest-likelihood topology for Neotominae. See Additional file 2: Figure S2 for details of the legend. Och = Ochrotomyini, Baiom = Baiomyini. Click here for file

Additional file 11 **Figure S11.** Cladogram depicting the highest-likelihood topology for Arvicolinae. See Additional file 2: Figure S2 for details of the legend. Pro = Prometheomyini, Dic = Dicrostonychini, Ond = Ondatrini, Plio = Pliomyini, Arv = Arvicolini, Ell = Ellobiusini, Lag = Lagurini. Click here for file

Additional file 12 **Figure S12.** Cladogram depicting the highest-likelihood topology for Murinae [part 1]. See Additional file 2: Figure S2 for details of the legend. Mic = Micromys division, Cru = Crunomys division, Max = Maxomys division, Mel = Melasmothrix division, Hydrom = Hydromyines division, Con = Conilurines division, Urom = Uromyines division. Click here for file

Additional file 13 **Figure S13.** Cladogram depicting the highest-likelihood topology for Murinae [part 2]. See Additional file 2: Figure S2 for details of the legend. Mic = Micromys division, Mil = Millardia division, Col = Colomys division, Cre = Cremnomys division, Gol = Golunda, Oen = Oenomys division, Hyb = Hybomys division, Mi = Micaelamys division, Das = Dasymys division, Mal = Malacomys division, Stenocep = Stenocephalemys division. Click here for file

Additional file 14 ML RaxML topology. Click here for file

Additional file 15 Rodentia species list names. Click here for file

Additional file 16 Rodentia accession numbers by gene. Click here for file

Additional file 17 Rodentia accession numbers by taxonomic group (Sheet 1: MYODONTA + ANOMALUROMORPHA; Sheet 2: SCIUROIDEA; Sheet 3: CTENOHYSTRICA; Sheet 4: CASTORIMORPHA). Click here for file

Additional file 18 Loci used in each molecular dating analysis (see Material and Methods). Click here for file
