# Supplementary figures and images for: A glimpse on the pattern of rodent diversification: a phylogenetic approach
Source: BMC Evol Biol. 2012 Jun 14;12:88. doi: 10.1186/1471-2148-12-88 (PMC3532383; doi:10.1186/1471-2148-12-88)

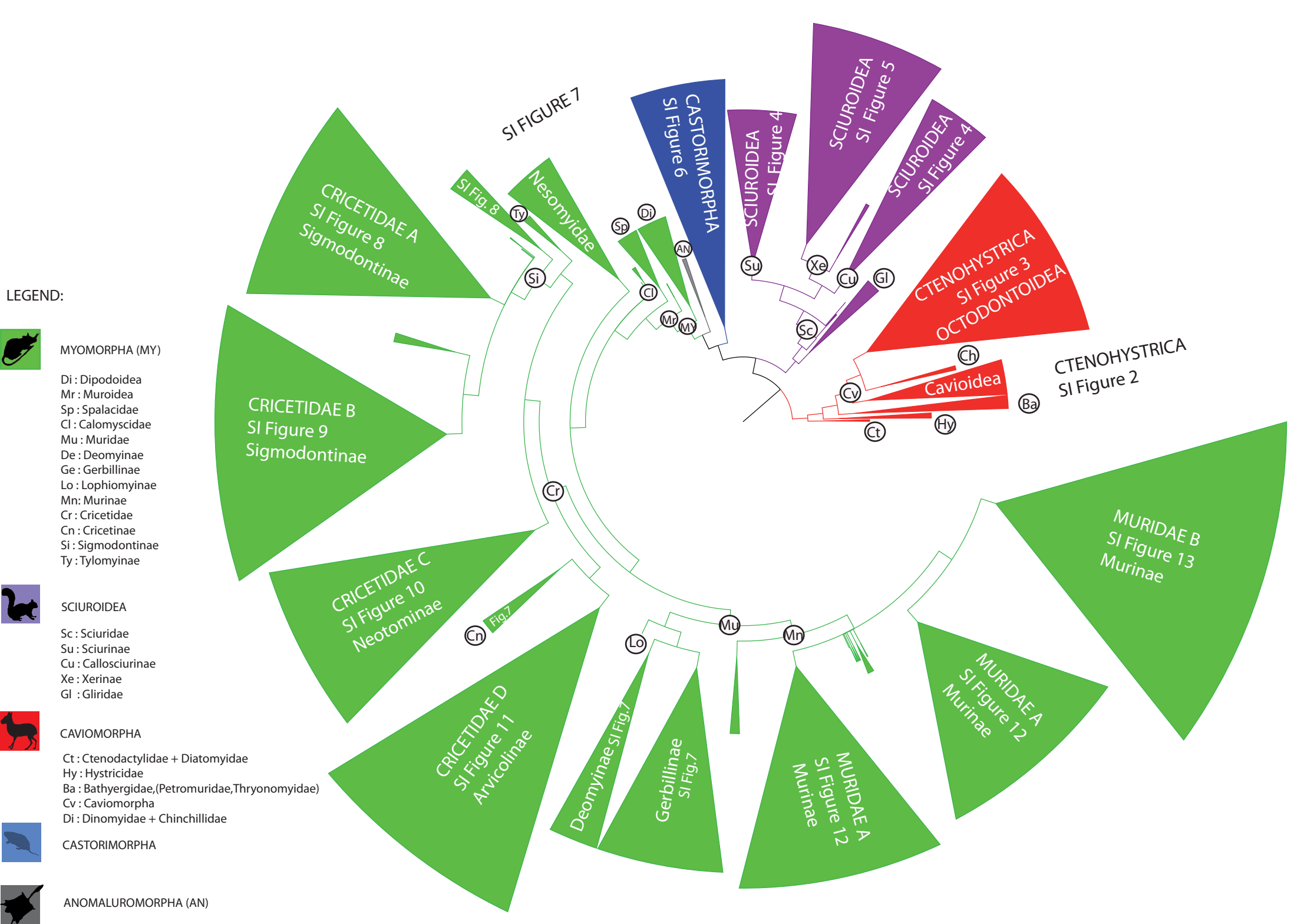

Supplement: Additional file 1 — Figure S1. Rodent species level evolutionary tree. Species-level phylogenetic topology based on the highest-likelihood tree inferred from the 11-gene supermatrix, and combined with the taxonomic information of Wilson and Reeder (2005). [file 1471-2148-12-88-S1.pdf]

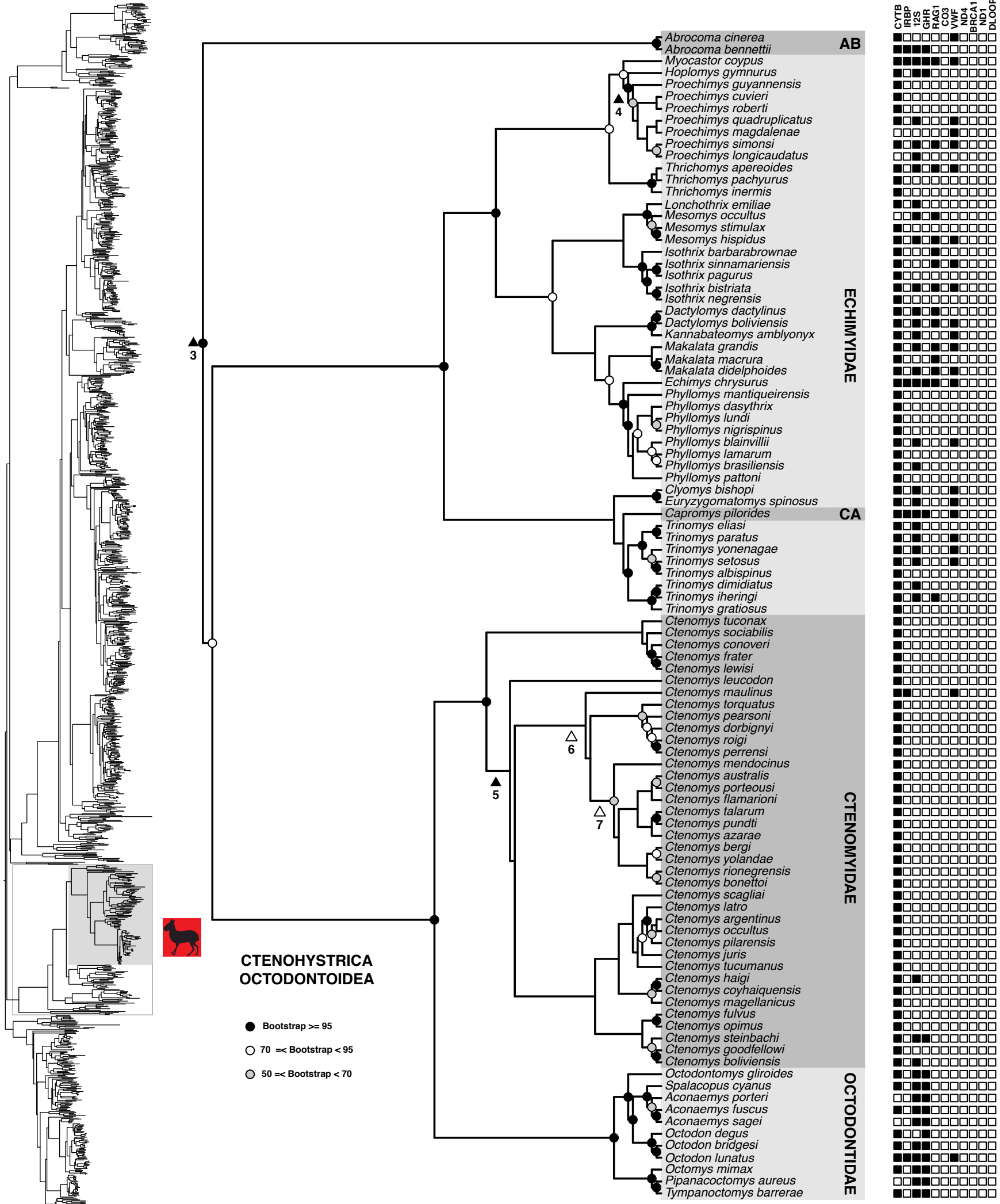

Supplement: Additional file 3 — Figure S3. Cladogram depicting the highest-likelihood topology for the Octodontoidea. See Additional file 2: Figure S2 for details of the legend. AB = Abrocomidae, CA = Capromyidae. [file 1471-2148-12-88-S3.pdf]

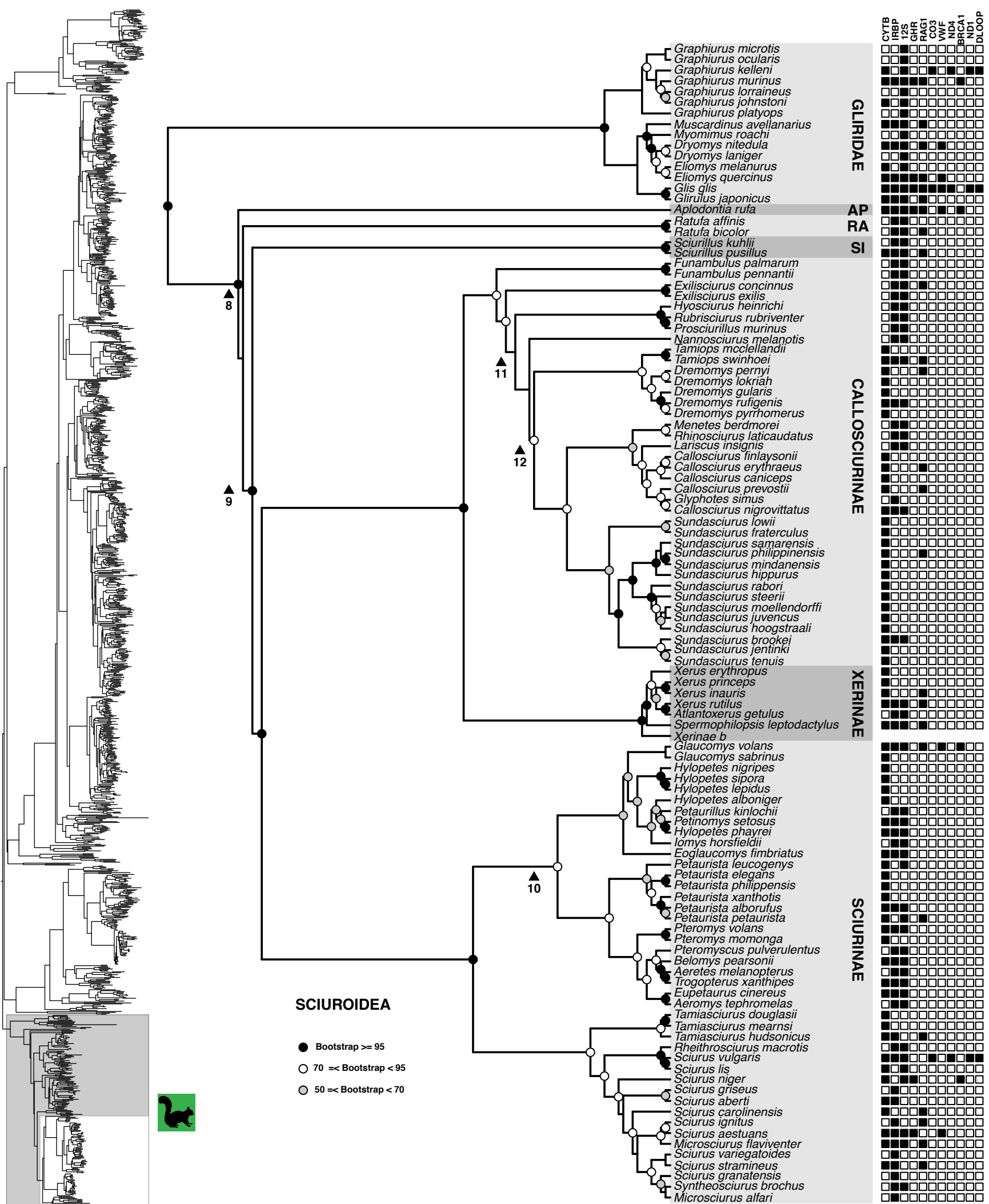

Supplement: Additional file 4 — Figure S4. Cladogram depicting the highest-likelihood topology for the Sciuroidea. See Additional file 2: Figure S2 for details of the legend. AP = Aplodontidae, RA = Ratufinae, SI = Sciurillinae. [file 1471-2148-12-88-S4.pdf]

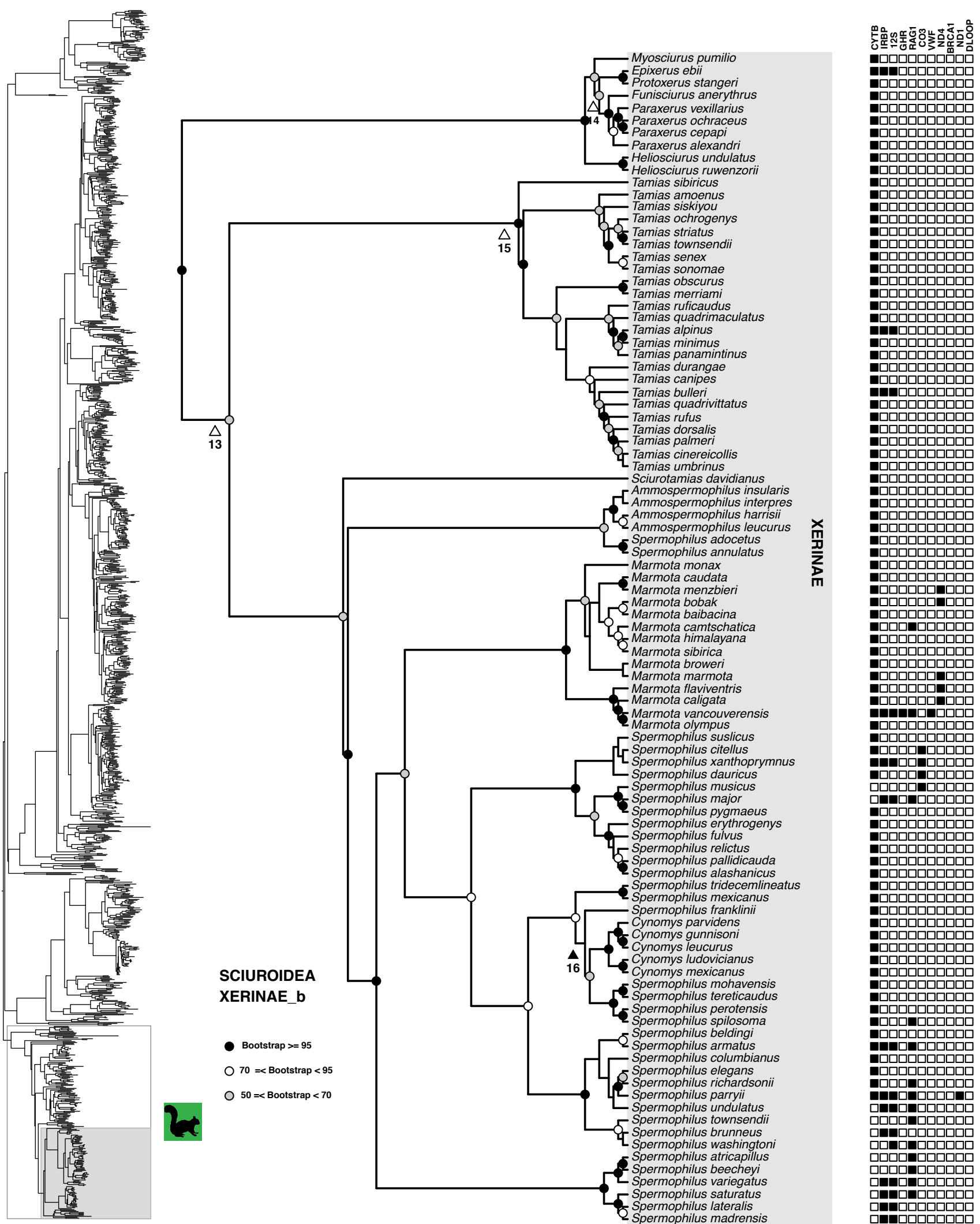

Supplement: Additional file 5 — Figure S5. Cladogram depicting the highest-likelihood topology for the Xerinae. See Additional file 2: Figure S2 for details of the legend. [file 1471-2148-12-88-S5.pdf]

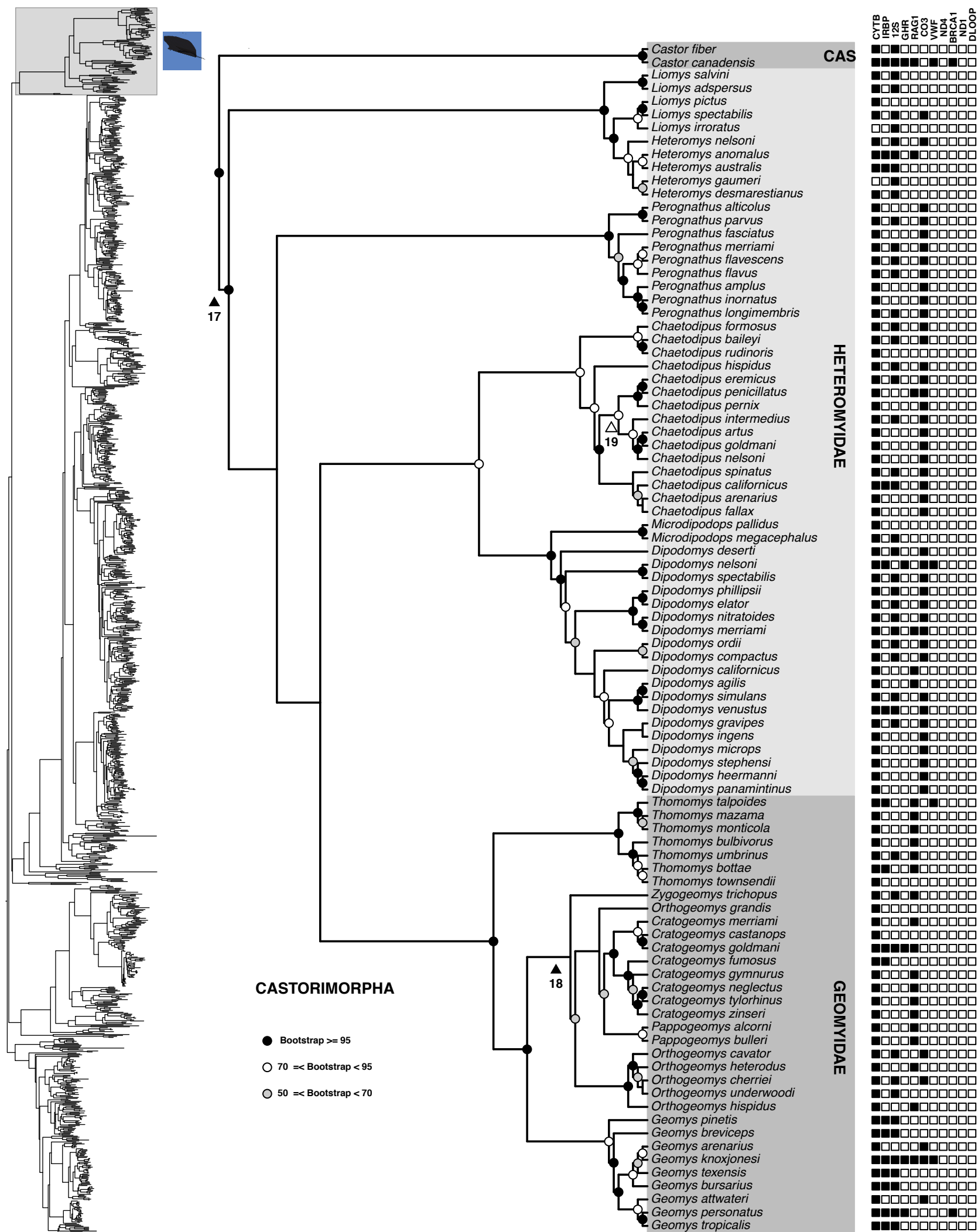

Supplement: Additional file 6 — Figure S6. Cladogram depicting the highest-likelihood topology for the Castorimorpha. See Additional file 2: Figure S2 for details of the legend. CAS = Castoridae. [file 1471-2148-12-88-S6.pdf]

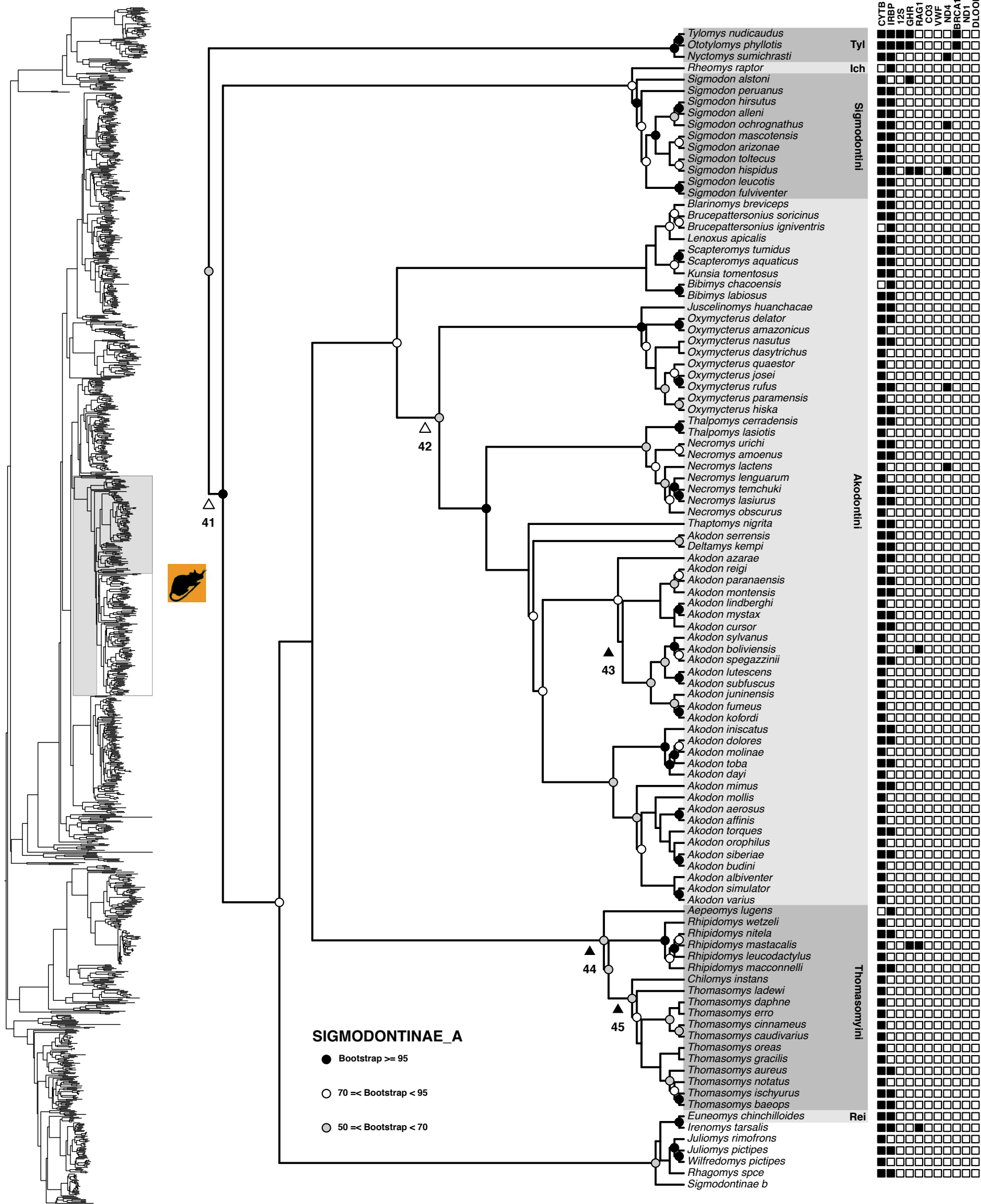

Supplement: Additional file 8 — Figure S8. Cladogram depicting the highest-likelihood topology for Sigmodontinae [part 1] + Tylomyinae. See Additional file 2: Figure S2 for details of the legend. Tyl = Tylomyinae, Ich = Ichthyomyini, Rei = Reithrodontini. [file 1471-2148-12-88-S8.pdf]

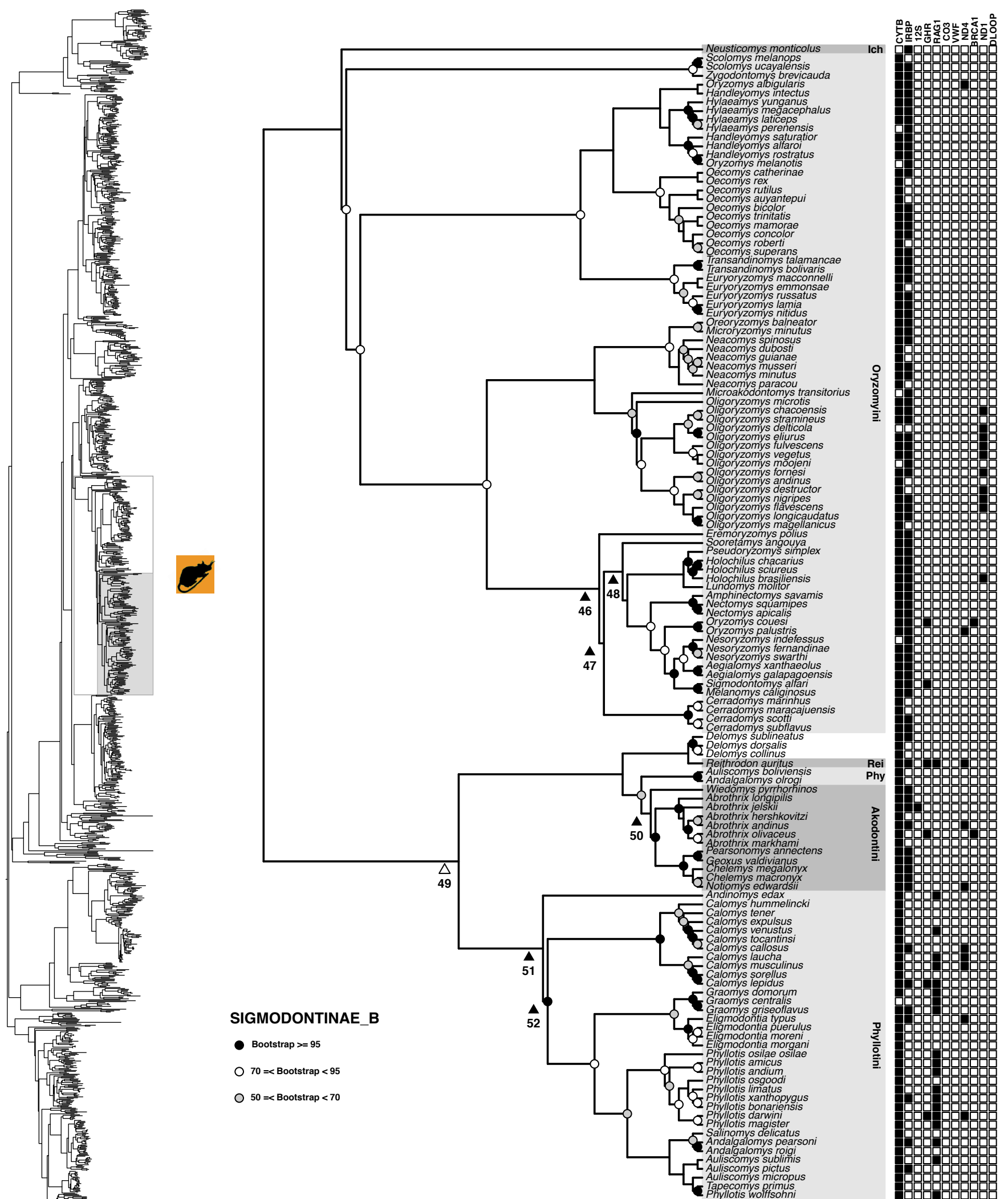

Supplement: Additional file 9 — Figure S9. Cladogram depicting the highest-likelihood topology for Sigmodontinae [part 2]. See Additional file 2: Figure S2 for details of the legend. Tyl = Tylomyinae, Ich = Ichthyomyini, Rei = Reithrodontini, Phy = Phyllotini. [file 1471-2148-12-88-S9.pdf]

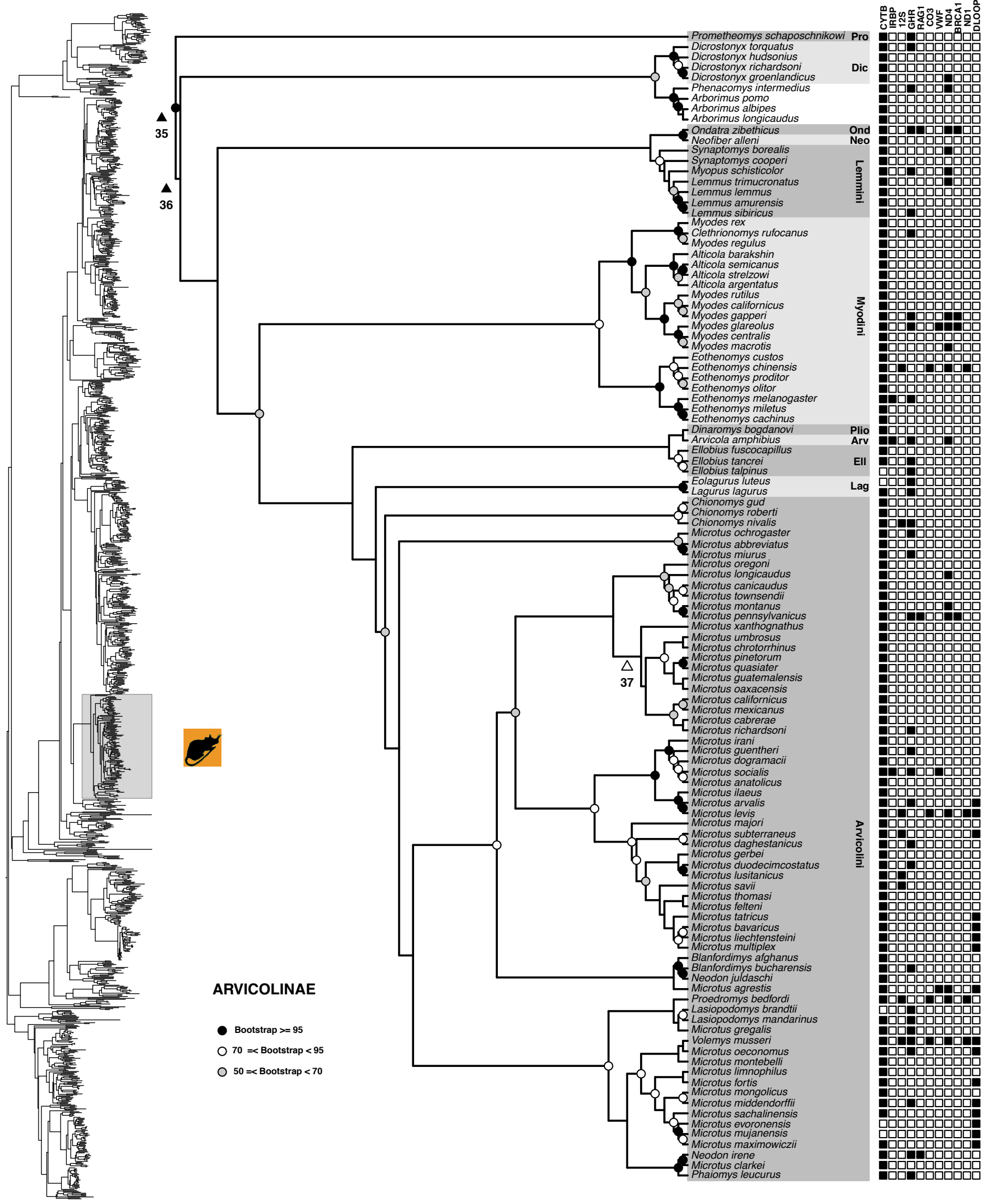

Supplement: Additional file 11 — Figure S11. Cladogram depicting the highest-likelihood topology for Arvicolinae. See Additional file 2: Figure S2 for details of the legend. Pro = Prometheomyini, Dic = Dicrostonychini, Ond = Ondatrini, Plio = Pliomyini, Arv = Arvicolini, Ell = Ellobiusini, Lag = Lagurini. [file 1471-2148-12-88-S11.pdf]

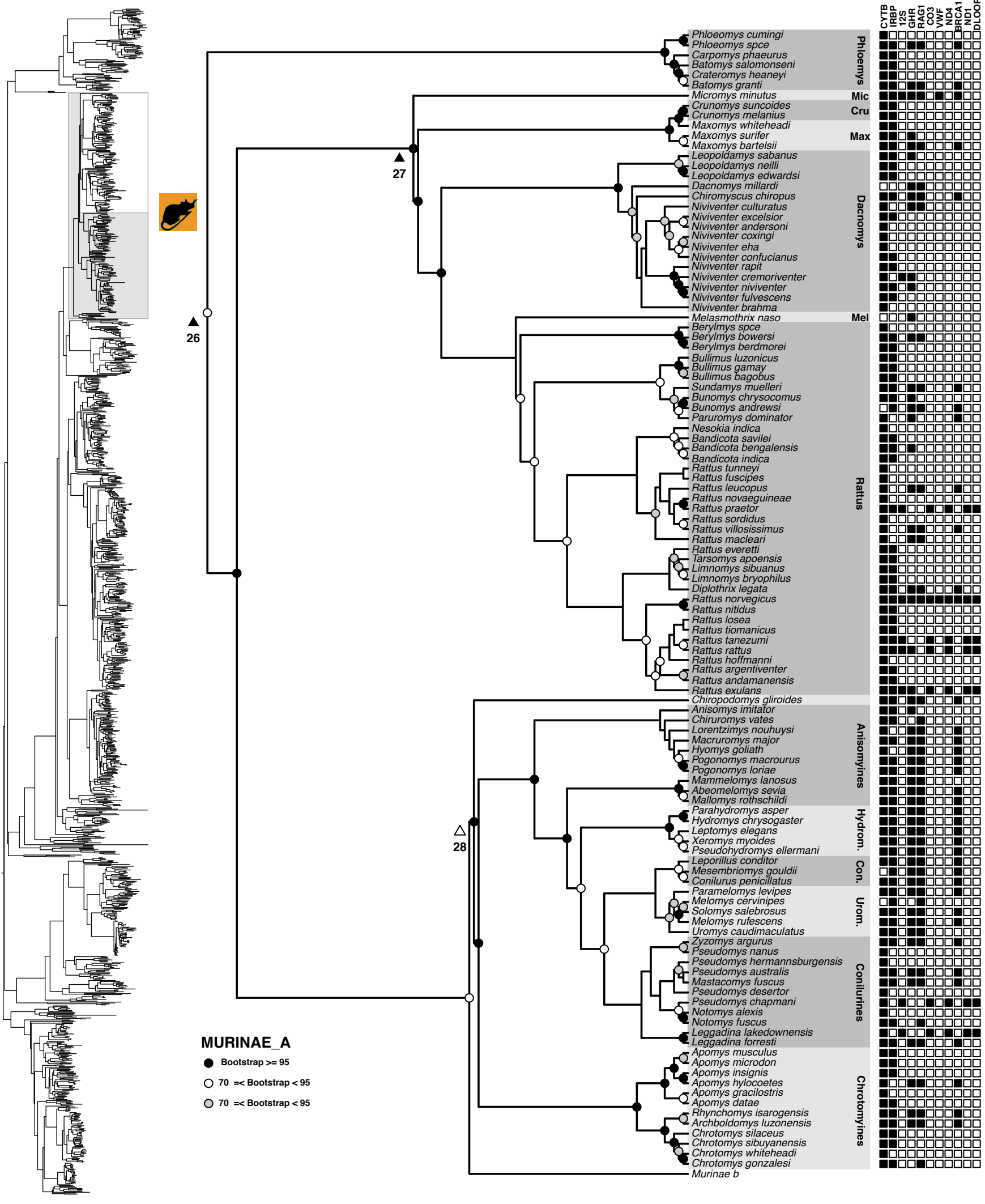

Supplement: Additional file 12 — Figure S12. Cladogram depicting the highest-likelihood topology for Murinae [part 1]. See Additional file 2: Figure S2 for details of the legend. Mic = Micromys division, Cru = Crunomys division, Max = Maxomys division, Mel = Melasmothrix division, Hydrom = Hydromyines division, Con = Conilurines division, Urom = Uromyines division. [file 1471-2148-12-88-S12.pdf]
