# Supplementary material for: A glimpse on the pattern of rodent diversification: a phylogenetic approach
Source: BMC Evol Biol. 2012 Jun 14;12:88. doi: 10.1186/1471-2148-12-88 (PMC3532383; doi:10.1186/1471-2148-12-88)
Supplement: Additional file 2 — Figure S2. Cladogram depicting the highest-likelihood topology for the Ctenohystrica. Circles at nodes represent bootstrap support (circles: black 100-95%, white 95-70%, gray 70-50%). Maximum likekihood tree (lnL=-714749.2). Displayed clade are highlighted using the simplified full ML topology on the left side of the figure. Molecular marker sampling is depicted for each taxa to the right of the tree. Names of the genes are given. Genes sampled for our dataset (Table 2) are marked in black square while missing genes are symbolized by white squares. Triangles indicate significant (P < 0.05) and marginal (0.05 < P < 0.1) shifts in diversification rate (SDR) as inferred by Δ1. LA = Diatomyidae, CT = Ctenodactylidae, TH = Thryonomyidae, PE = Petromuridae, CU = Cuniculidae, DASYPROC = Dasyproctidae, DI = Dinomyidae, CHINCHILLID = Chinchillidae. Bootstrap for all nodes and topology can be found in Additional file 14. [file 1471-2148-12-88-S2.pdf]

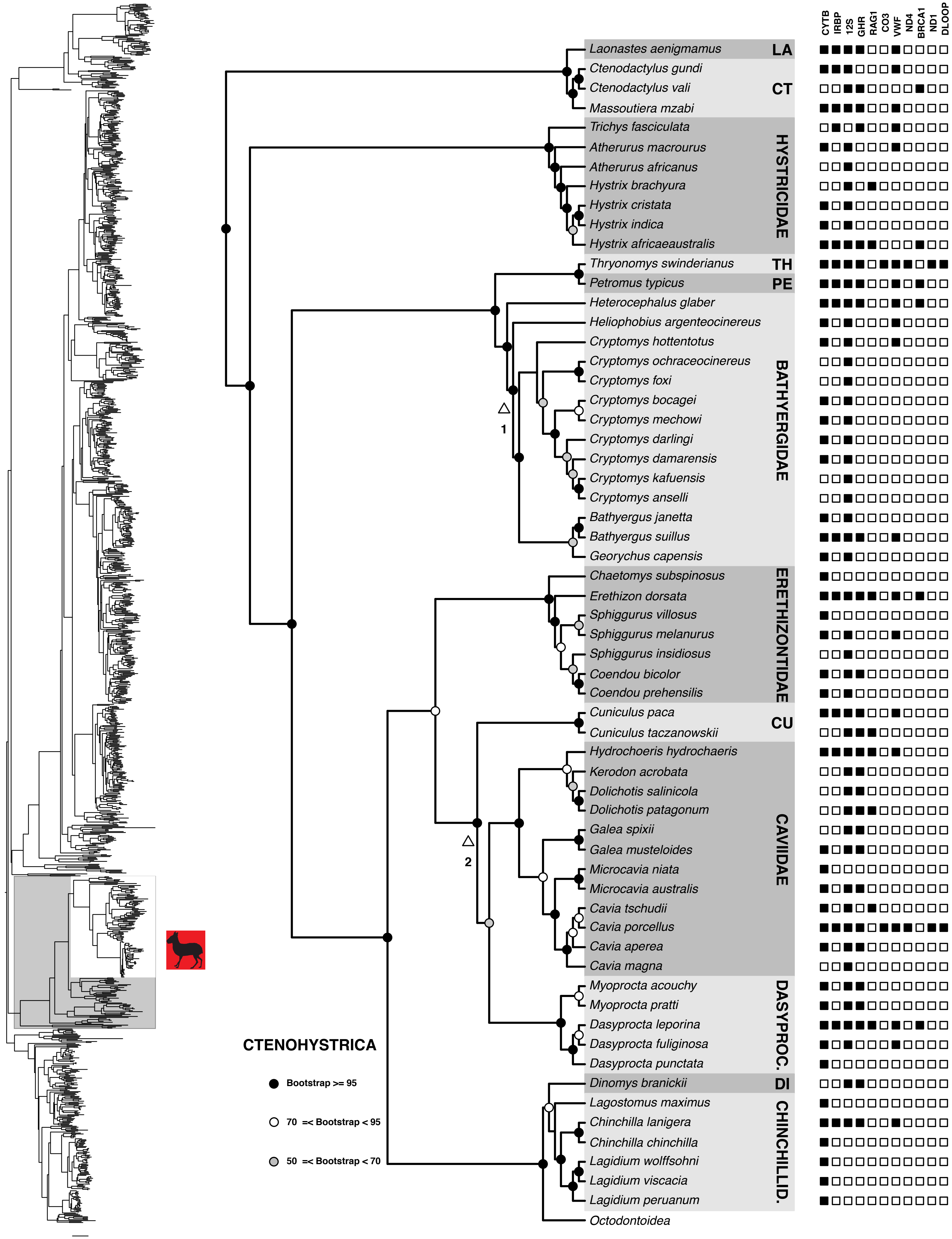
CYTB
IRBP
12S
GHR
RAG1
CO3
VWF
ND4
BRCA1
ND1
DLOOP
Laonastes aenigmamus
LA
Ctenodactylus gundi
Ctenodactylus vali
CT
Massoutiera mzabi
Trichys fasciculata
Atherurus macrourus
Atherurus africanus
Hystrix brachyura
Hystrix cristata
Hystrix indica
Hystrix africaeaustralis
HYSTRICIDAE
Thryonomys swinderianus
TH
Petromus typicus
PE
Heterocephalus glaber
Heliophobius argenteocinereus
Cryptomys hottentotus
Cryptomys ochraceocinereus
Cryptomys foxi
Cryptomys bocagei
Cryptomys mechowi
Cryptomys darlingi
Cryptomys damarensis
Cryptomys kafuensis
Cryptomys anselli
Bathyergus janetta
Bathyergus suillus
Georychus capensis
BATHYERGIDAE
1
Chaetomys subspinosus
Erethizon dorsata
Sphiggurus villosus
Sphiggurus melanurus
Sphiggurus insidiosus
Coendou bicolor
Coendou prehensilis
ERETHIZONTIDAE
Cuniculus paca
Cuniculus taczanowskii
CU
Hydrochoeris hydrochaeris
Kerodon acrobata
Dolichotis salinicola
Dolichotis patagonum
Galea spixii
Galea musteloides
Microcavia niata
Microcavia australis
Cavia tschudii
Cavia porcellus
Cavia aperea
Cavia magna
CAVIIDAE
2
Myoprocta acouchy
Myoprocta pratti
Dasyprocta leporina
Dasyprocta fuliginosa
Dasyprocta punctata
DASYPROC.
Dinomys branickii
DI
Lagostomus maximus
Chinchilla lanigera
Chinchilla chinchilla
Lagidium wolffsohni
Lagidium viscacia
Lagidium peruanum
CHINCHILLID.
Octodontoidea
CTENOHYSTRICA
Bootstrap >= 95
70 =< Bootstrap < 95
50 =< Bootstrap < 70
