# Supplementary material for: A glimpse on the pattern of rodent diversification: a phylogenetic approach
Source: BMC Evol Biol. 2012 Jun 14;12:88. doi: 10.1186/1471-2148-12-88 (PMC3532383; doi:10.1186/1471-2148-12-88)
Supplement: Additional file 7 — Figure S7. Cladogram depicting the highest-likelihood topology for the mouse-related clade. See Additional file 2: Figure S2 for details of the legend. PED = Pedetidae, ANO = Anomaluroidea, PLA = Platacanthomyidae, Rhy = Rhyzomyidae, Spalac = Spalacidae, CAL = Calomyscidae, LOP = Lophiomyinae. [file 1471-2148-12-88-S7.pdf]

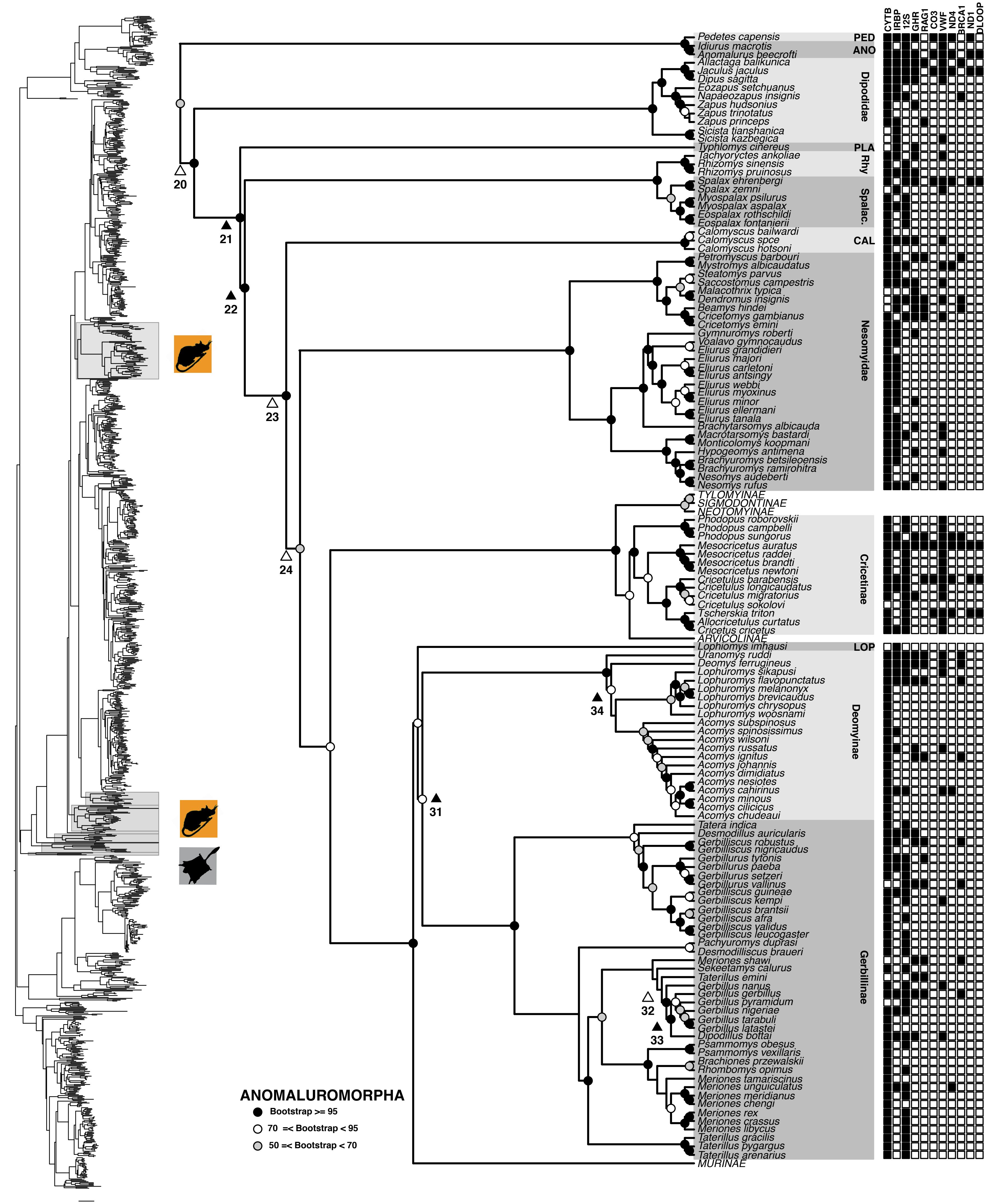
CYTB
IRBP
12S
GHR
RAG1
CO3
VWF
ND4
BRCA1
ND1
DLOOP
Pedetes capensis
Idiurus macrotis
Anomalurus beecrofti
Allactaga balikunica
Jaculus jaculus
Dipus sagitta
Eozapus setchuanus
Napaeozapus insignis
Zapus hudsonius
Zapus trinotatus
Zapus princeps
Sicista tianshanica
Sicista kazbegica
Typhlomys cinereus
Tachyoryctes ankoliae
Rhizomys sinensis
Rhizomys pruinosus
Spalax ehrenbergi
Spalax zemni
Myospalax psilurus
Myospalax aspalax
Eospalax rothschildi
Eospalax fontanierii
Calomyscus bailwardi
Calomyscus spce
Calomyscus hotsoni
Petromyscus barbouri
Mystromys albicaudatus
Steatomys parvus
Saccostomus campestris
Malacothrix typica
Dendromus insignis
Beamys hindei
Cricetomys gambianus
Cricetomys emini
Gymnuromys roberti
Voalavo gymnocaudus
Eliurus grandidieri
Eliurus majori
Eliurus carletoni
Eliurus antsingy
Eliurus webbi
Eliurus myoxinus
Eliurus minor
Eliurus ellermani
Eliurus tanala
Brachytarsomys albicauda
Macrotarsomys bastardi
Monticolomys koopmani
Hypogeomys antimena
Brachyuromys betsileoensis
Brachyuromys ramirohitra
Nesomys audeberti
Nesomys rufus
TYLOMYINAE
SIGMODONTINAE
NEOTOMYINAE
Phodopus roborovskii
Phodopus campbelli
Phodopus sungorus
Mesocricetus auratus
Mesocricetus raddei
Mesocricetus brandti
Mesocricetus newtoni
Cricetulus barabensis
Cricetulus longicaudatus
Cricetulus migratorius
Cricetulus sokolovi
Tscherskia triton
Allocricetulus curtatus
Cricetus cricetus
ARVICOLINAE
Lophiomys imhausi
Uranomys ruddi
Deomys ferrugineus
Lophuromys sikapusi
Lophuromys flavopunctatus
Lophuromys melanonyx
Lophuromys brevicaudus
Lophuromys chrysopus
Lophuromys woosnami
Acomys subspinosus
Acomys spinosissimus
Acomys wilsoni
Acomys russatus
Acomys ignitus
Acomys johannis
Acomys dimidiatus
Acomys nesiotes
Acomys cahirinus
Acomys minous
Acomys cilicicus
Acomys chudeaui
Tatera indica
Desmodillus auricularis
Gerbilliscus robustus
Gerbilliscus nigricaudus
Gerbillurus tytonis
Gerbillurus paeba
Gerbillurus setzeri
Gerbillurus vallinus
Gerbilliscus guineae
Gerbilliscus kempi
Gerbilliscus brantsii
Gerbilliscus afra
Gerbilliscus validus
Gerbilliscus leucogaster
Pachyuromys duprasi
Desmodilliscus braueri
Meriones shawi
Sekeetamys calurus
Taterillus emini
Gerbillus nanus
Gerbillus gerbillus
Gerbillus pyramidum
Gerbillus nigeriae
Gerbillus tarabuli
Gerbillus latastei
Dipodillus bottai
Psammomys obesus
Psammomys vexillaris
Brachiones przewalskii
Rhombomys opimus
Meriones tamariscinus
Meriones unguiculatus
Meriones meridianus
Meriones chengi
Meriones rex
Meriones crassus
Meriones libycus
Taterillus gracilis
Taterillus pygargus
Taterillus arenarius
MURINAE
PED
ANO
Dipodidae
PLA
Rhy
Spalac.
CAL
Nesomyidae
Cricetinae
LOP
Deomyinae
Gerbillinae
20
21
22
23
24
31
32
33
34
ANOMALUROMORPHA
Bootstrap >= 95
70 =< Bootstrap < 95
50 =< Bootstrap < 70
