# Supplementary material for: A glimpse on the pattern of rodent diversification: a phylogenetic approach
Source: BMC Evol Biol. 2012 Jun 14;12:88. doi: 10.1186/1471-2148-12-88 (PMC3532383; doi:10.1186/1471-2148-12-88)
Supplement: Additional file 10 — Figure S10. Cladogram depicting the highest-likelihood topology for Neotominae. See Additional file 2: Figure S2 for details of the legend. Och = Ochrotomyini, Baiom = Baiomyini. [file 1471-2148-12-88-S10.pdf]

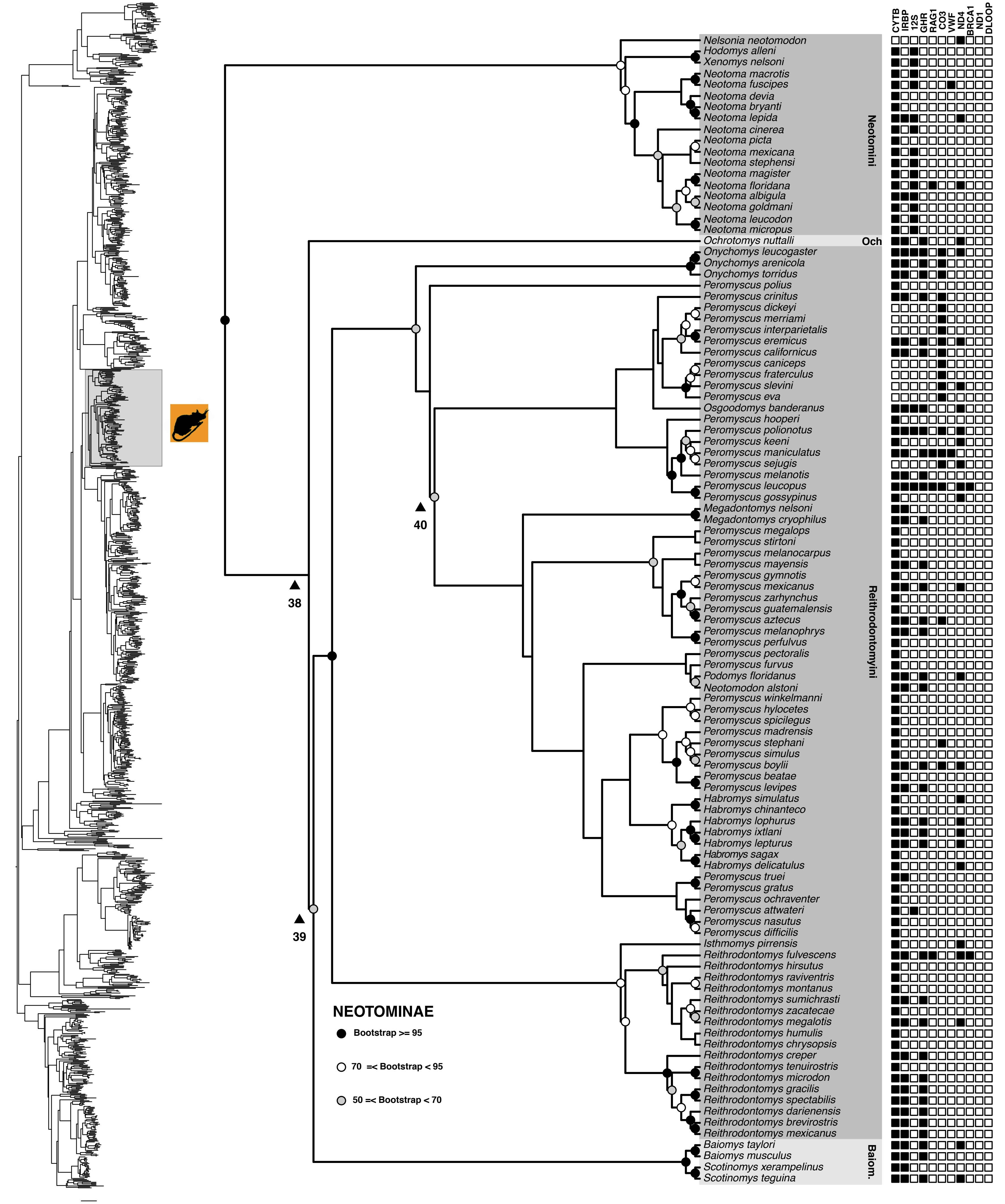
CYTB
IRBP
12S
GHR
RAG1
CO3
VWF
ND4
BRCA1
ND1
DLOOP
Nelsonia neotomodon
Hodomys alleni
Xenomys nelsoni
Neotoma macrotis
Neotoma fuscipes
Neotoma devia
Neotoma bryanti
Neotoma lepida
Neotoma cinerea
Neotoma picta
Neotoma mexicana
Neotoma stephensi
Neotoma magister
Neotoma floridana
Neotoma albigula
Neotoma goldmani
Neotoma leucodon
Neotoma micropus
Neotomini
Ochrotomys nuttalli
Och
Onychomys leucogaster
Onychomys arenicola
Onychomys torridus
Peromyscus polius
Peromyscus crinitus
Peromyscus dickeyi
Peromyscus merriami
Peromyscus interparietalis
Peromyscus eremicus
Peromyscus californicus
Peromyscus caniceps
Peromyscus fraterculus
Peromyscus slevini
Peromyscus eva
Osgoodomys banderanus
Peromyscus hooperi
Peromyscus polionotus
Peromyscus keeni
Peromyscus maniculatus
Peromyscus sejugis
Peromyscus melanotis
Peromyscus leucopus
Peromyscus gossypinus
Megadontomys nelsoni
Megadontomys cryophilus
Peromyscus megalops
Peromyscus stirtoni
Peromyscus melanocarpus
Peromyscus mayensis
Peromyscus gymnotis
Peromyscus mexicanus
Peromyscus zarhynchus
Peromyscus guatemalensis
Peromyscus aztecus
Peromyscus melanophrys
Peromyscus perfulvus
Peromyscus pectoralis
Peromyscus furvus
Podomys floridanus
Neotomodon alstoni
Peromyscus winkelmanni
Peromyscus hylocetes
Peromyscus spicilegus
Peromyscus madrensis
Peromyscus stephani
Peromyscus simulus
Peromyscus boylii
Peromyscus beatae
Peromyscus levipes
Habromys simulatus
Habromys chinanteco
Habromys lophurus
Habromys ixtlani
Habromys lepturus
Habromys sagax
Habromys delicatulus
Peromyscus truei
Peromyscus gratus
Peromyscus ochraventer
Peromyscus attwateri
Peromyscus nasutus
Peromyscus difficilis
Isthmomys pirrensis
Reithrodontomys fulvescens
Reithrodontomys hirsutus
Reithrodontomys raviventris
Reithrodontomys montanus
Reithrodontomys sumichrasti
Reithrodontomys zacatecae
Reithrodontomys megalotis
Reithrodontomys humulis
Reithrodontomys chrysopsis
Reithrodontomys creper
Reithrodontomys tenuirostris
Reithrodontomys microdon
Reithrodontomys gracilis
Reithrodontomys spectabilis
Reithrodontomys darienensis
Reithrodontomys brevirostris
Reithrodontomys mexicanus
Reithrodontomyini
Baiomys taylori
Baiomys musculus
Scotinomys xerampelinus
Scotinomys teguina
Baiom.
38
39
40
NEOTOMINAE
Bootstrap >= 95
70 =< Bootstrap < 95
50 =< Bootstrap < 70
