# Supplementary material for: A glimpse on the pattern of rodent diversification: a phylogenetic approach
Source: BMC Evol Biol. 2012 Jun 14;12:88. doi: 10.1186/1471-2148-12-88 (PMC3532383; doi:10.1186/1471-2148-12-88)
Supplement: Additional file 13 — Figure S13. Cladogram depicting the highest-likelihood topology for Murinae [part 2]. See Additional file 2: Figure S2 for details of the legend. Mic = Micromys division, Mil = Millardia division, Col = Colomys division, Cre = Cremnomys division, Gol = Golunda, Oen = Oenomys division, Hyb = Hybomys division, Mi = Micaelamys division, Das = Dasymys division, Mal = Malacomys division, Stenocep = Stenocephalemys division. [file 1471-2148-12-88-S13.pdf]

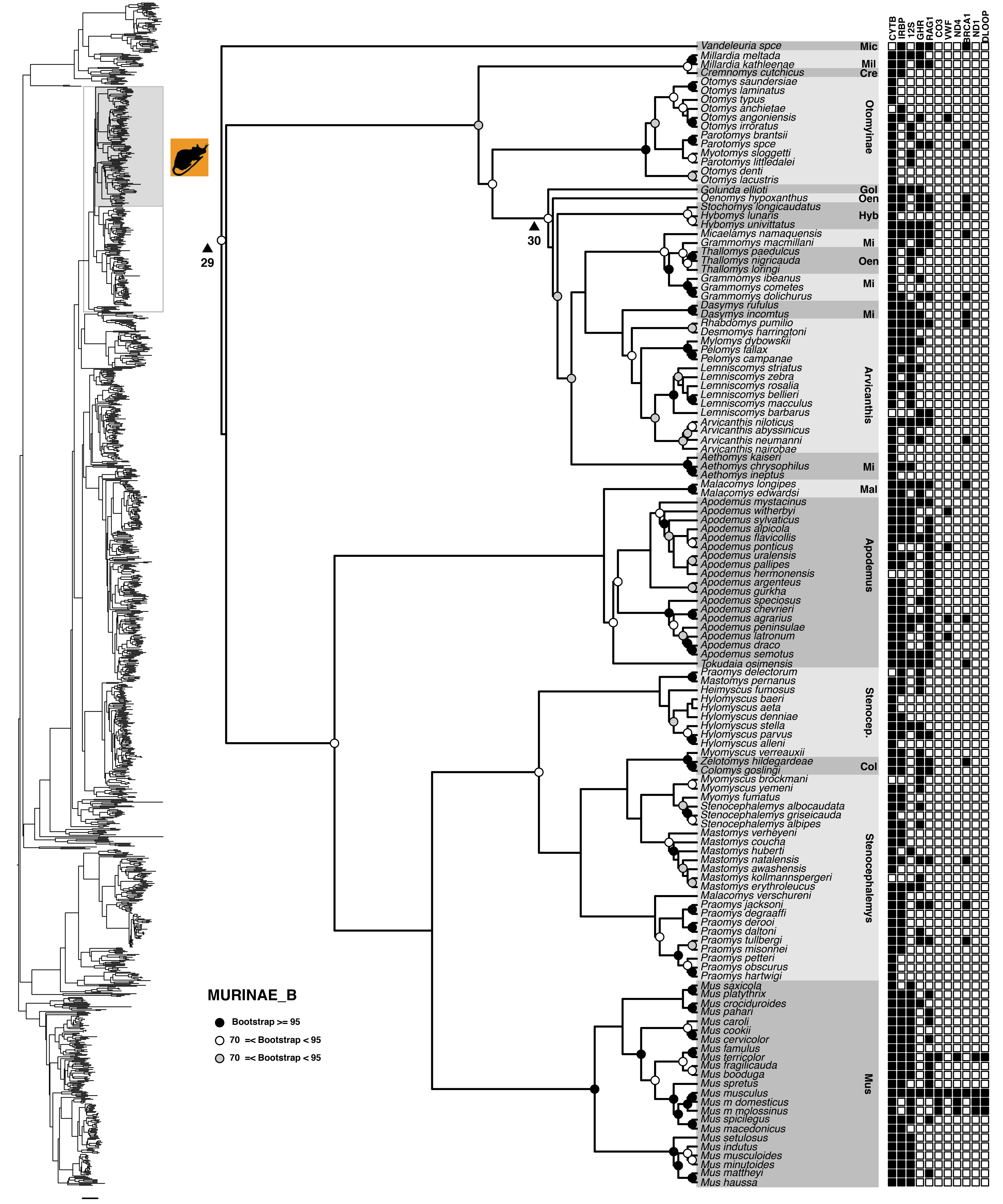
CYTB
IRBP
12S
GHR
RAG1
CO3
VWF
ND4
BRCA1
ND1
DLOOP
29
30
MURINAE_B
Bootstrap >= 95
70 =< Bootstrap < 95
70 =< Bootstrap < 95
Vandeleuria spce
Mic
Millardia meltada
Millardia kathleenae
Mil
Cremnomys cutchicus
Cre
Otomys saundersiae
Otomys laminatus
Otomys typus
Otomys anchietae
Otomys angoniensis
Otomys irroratus
Parotomys brantsii
Parotomys spce
Myotomys sloggetti
Parotomys littledalei
Otomys denti
Otomys lacustris
Otomyinae
Golunda ellioti
Gol
Oenomys hypoxanthus
Oen
Stochomys longicaudatus
Hybomys lunaris
Hybomys univittatus
Hyb
Micaelamys namaquensis
Grammomys macmillani
Mi
Thallomys paedulcus
Thallomys nigricauda
Thallomys loringi
Oen
Grammomys ibeanus
Grammomys cometes
Grammomys dolichurus
Mi
Dasymys rufulus
Dasymys incomtus
Mi
Rhabdomys pumilio
Desmomys harringtoni
Mylomys dybowskii
Pelomys fallax
Pelomys campanae
Lemniscomys striatus
Lemniscomys zebra
Lemniscomys rosalia
Lemniscomys bellieri
Lemniscomys macculus
Lemniscomys barbarus
Arvicanthis niloticus
Arvicanthis abyssinicus
Arvicanthis neumanni
Arvicanthis nairobae
Arvicanthis
Aethomys kaiseri
Aethomys chrysophilus
Aethomys ineptus
Mi
Malacomys longipes
Malacomys edwardsi
Mal
Apodemus mystacinus
Apodemus witherbyi
Apodemus sylvaticus
Apodemus alpicola
Apodemus flavicollis
Apodemus ponticus
Apodemus uralensis
Apodemus pallipes
Apodemus hermonensis
Apodemus argenteus
Apodemus gurkha
Apodemus speciosus
Apodemus chevrieri
Apodemus agrarius
Apodemus peninsulae
Apodemus latronum
Apodemus draco
Apodemus semotus
Tokudaia osimensis
Apodemus
Praomys delectorum
Mastomys pernanus
Heimyscus fumosus
Hylomyscus baeri
Hylomyscus aeta
Hylomyscus denniae
Hylomyscus stella
Hylomyscus parvus
Hylomyscus alleni
Myomyscus verreauxii
Stenocep.
Zelotomys hildegardeae
Colomys goslingi
Col
Myomyscus brockmani
Myomyscus yemeni
Myomys fumatus
Stenocephalemys albocaudata
Stenocephalemys griseicauda
Stenocephalemys albipes
Mastomys verheyeni
Mastomys coucha
Mastomys huberti
Mastomys natalensis
Mastomys awashensis
Mastomys kollmannspergeri
Mastomys erythroleucus
Malacomys verschureni
Praomys jacksoni
Praomys degraaffi
Praomys derooi
Praomys daltoni
Praomys tullbergi
Praomys misonnei
Praomys petteri
Praomys obscurus
Praomys hartwigi
Stenocephalemys
Mus saxicola
Mus platythrix
Mus crociduroides
Mus pahari
Mus caroli
Mus cookii
Mus cervicolor
Mus famulus
Mus terricolor
Mus fragilicauda
Mus booduga
Mus spretus
Mus musculus
Mus m domesticus
Mus m molossinus
Mus spicilegus
Mus macedonicus
Mus setulosus
Mus indutus
Mus musculoides
Mus minutoides
Mus mattheyi
Mus haussa
Mus
